# Sociodemographic and clinical characteristics associated with health literacy in people hospitalized for chronic diseases*

**DOI:** 10.1590/1518-8345.7395.4494

**Published:** 2025-05-02

**Authors:** Francini de Oliveira Rodrigues, Eliana Elisa Rehfeld Gheno, Letícia Y Castro, Arnaldo Nogaro, Amanda Caroline Mélo da Rosa, Adriane Cristina Bernat Kolankiewicz

**Affiliations:** 1 Universidade Regional do Noroeste do Estado do Rio Grande do Sul, Ijuí, RS, Brazil; 2 Universidade do Alto Uruguai e das Missões, Campus Erechim, RS, Brazil; 3 Scholarship holder at the Conselho Nacional de Desenvolvimento Científico e Tecnológico (CNPq), Brazil

**Keywords:** Health Literacy, Noncommunicable Diseases, Hospitalization, Chronic Diseases, Access to Information, Health Management

## Abstract

to identify the association between health literacy and sociodemographic and clinical variables of hospitalized patients in people hospitalized with chronic diseases.

this was a cross-sectional study of patients with chronic illnesses admitted to a general hospital. A sociodemographic/clinical questionnaire and the Health Literacy Questionnaire comprising nine scales measured by scores, were used, with descriptive and inferential data analysis.

Social Support for Health was associated with the level of primary education (p=0.009). Understanding and Support from Health Professionals were related to the length of chronic illness (p=0.044). Evaluation of Health Information was associated with age (p=0.001), being able to read (p=0.010) and write (p=0.032). Navigating the Health System was also associated with age (p=0.018), as was the Ability to Find Good Health Information (p=0.002) and being able to read (p=0.010), and Understanding Health Information Well Enough to Know What to Do was associated with age (p=0.001) and being able to write (p=0.010).

schooling, age group, length of time with a chronic illness, and being able to read and write interfere with health literacy. This highlights the need for personalized strategies that take these variables into account in order to improve health literacy in hospitalized populations.

## Highlights

**(1)** The first Brazilian study to assess the health literacy of people with CNCDs. **(2)** Strengths: ability to interact, navigate the system, and social support. **(3)** Weaknesses: evaluation of health information and ability to find it. **(4)** Age, schooling, and ability to read and write interfere with health literacy.

## Introduction

Chronic Non-Communicable Diseases (CNCDs) are a global public health problem. They are the main cause of disability and premature mortality in the world and are responsible for the deaths of 40.8 million people, equivalent to 74% of all deaths ^( 1 )^.

Being affected by CNCDs implies access to public goods and services, guaranteed rights, information, employment, income, and the possibility of making choices that are favorable to health ^( 2 )^. In this context, Health Literacy (HL) contributes to and can modify negative health factors and help understand individuals’ and communities’ strengths and weaknesses. It represents the knowledge and personal skills that are accumulated through daily activities, social interactions, and generations. Mediated by organizational structures and the availability of resources that enable people to access, understand, evaluate, and use information and services in ways that promote and maintain good health and well-being ^( 3 )^.

HL is considered to be the best indicator of health concerning many social determinants ^( 4 )^. Insufficient HL results in inappropriate use of services and generates negative health outcomes. It is also associated with high hospitalization rates, an increase in the prevalence of diseases, less use of preventive methods, and low adherence to treatment. On the other hand, when appropriate, it improves self-management of care and Quality of Life (QoL) and, consequently, reduces hospital readmissions, burdens, and costs ^( 5 )^.

In this context, patients whose health is worsened by CNCDs often require hospitalization due to their clinical complexity. And the health professionals who care for them, especially nurses, need to help prepare them for discharge safely and in a way that provides continuity of care. To this end, it is necessary to build skills, considering the level of HL, to facilitate the management, empowerment, and understanding of self-care, and consequently avoid adverse events and readmissions ^( 6 )^.

Some studies have limited themselves to assessing cognitive reading and numeracy skills, without considering the other aspects of HL that allow individuals and society to be responsible for their health conditions ^( 7 )^. A survey of people affected by CNCDs in Ethiopia found that HL levels vary according to sociodemographic and clinical characteristics. In this sense, health professionals should assess the level of HL and adapt information and support to HL skills according to the patient’s context, which favors the management of their chronic condition throughout life ^( 8 )^.

In the international context, other studies have identified the relationship between HL and sociodemographic characterization ^( 9 )^, but not with clinical conditions. In Brazil, some studies have been carried out to assess multidimensional HL in different populations, such as health professionals, users in general, home caregivers, and people with the Human Immunodeficiency Virus (HIV) ^( 10 - 12 )^. In Brazil, HL is little explored in the context of health practices and management, and there is no standardization of effective strategies and plans for better HL and its effectiveness. Above all, these can be applied during the hospitalization period, since few institutions carry out actions focused on them so that they can contribute to reducing hospital readmissions.

Thus, by identifying the associations between HL and sociodemographic and clinical variables, nursing professionals and health institutions will be able to recognize the weaknesses of HL and develop effective strategies for people with chronic diseases in order to reduce readmissions. There is a scarcity of national and international studies relating HL to sociodemographic and clinical characterization from the perspective of people living with CNCDs. Given this context and the knowledge gap identified, this study is justified.

Thus, this study aims to identify the association between HL and sociodemographic and clinical variables in patients hospitalized with chronic diseases.

## Method

### Type of study

This is a quantitative, cross-sectional study based on the Strengthening the Reporting of Observational Studies in Epidemiology (STROBE) guidelines. It should be noted that HL in the hospital context will be analyzed from the perspective of patients with chronic illnesses, based on sociodemographic and clinical variables.

### Context and data collection

The study was carried out in a general hospital with 76 beds in clinical inpatient units in the city of Ijuí (RS), Brazil. The units consist of 18 private beds and 58 double non-private beds. The institution has a “Preventive Medicine in Comprehensive Health Care” program, which accompanies patients through comprehensive care activities and preparation for hospital discharge.

### Period

Data was collected between April and October 2023.

### Participants

166 people hospitalized with CNCDs took part in the study.

### Selection criteria

Inclusion criteria: patients over the age of 18, with a diagnosis of CNCD recorded in their medical records, and hospitalized for at least 24 hours. Excluded were parturients, surgical patients, institutionalized patients after discharge, and people with non-preserved cognitive activity, which was assessed by the first author, a nurse, via records in the medical chart and in person at the bedside, based on an assessment of the patient’s level of consciousness, as well as the content.

### Sample definition

The study population consisted of patients admitted to the institution with CNCDs between April and August 2023, which reached a population size of approximately 370 patients, which characterizes a finite population. The main study factor, the Health Literacy Questionnaire (HLQ) scale, was taken as the basis for calculating the sample size. For the total scale, an average score of 3.0 (±1.0) was assumed, which defines the midpoint.

Based on this estimate and assuming a significance level of 5% (α=0.05), a margin of error of 7.5% on the mean to be estimated, which is equivalent to a variation of 0.2 units, the minimum sample size was set at 159 patients. Applying the correction factor for finite populations, the minimum size required for this study was estimated at 112 patients. Therefore, since 166 patients were investigated, a sample size that validated the sample’s representativeness was met.

Electronic medical records were analyzed daily to identify the patient’s clinical profile and length of stay, and they were invited during the data collection period. Of the medical records analyzed, 192 patients were invited to take part in the study, and, of these, 26 refused. Thus, the sample consisted of 166 patients hospitalized with NCDs.

### Study variables

The sociodemographic variables considered were age, gender, income, schooling, race, marital status, being part of the Preventive Medicine program, being able to read and write. And clinical variables such as: health conditions, time since diagnosis, having one or more chronic diseases. For the LS, the scales of the HLQ questionnaire were used.

## Instruments used to collect information

Information on the patients’ clinical conditions was obtained from the health records. For the sociodemographic and HL variables, a sociodemographic questionnaire and the HLQ were used, respectively, at the bedside. The HLQ is a multidimensional instrument for measuring HL ^( 7 )^, validated for use in Brazil ^( 10 )^.

The HLQ comprises 44 items, subdivided into two parts. Part 1 includes the following scales: 1. Feeling Understood and Supported by Healthcare Providers (HPS); 2. Having Sufficient Information to Manage One’s Health (HIS); 3. Active Health Care (AMH) 4. Social Support for Health (SS) 5. Appraisal of Health Information (CA) In these scales, the score for calculating the average is one to four, with answers ranging from “totally disagree” to “totally agree” ^( 7 , 10 )^.

Part 2 contains the following scales: 6. Ability to Actively Engage with Healthcare Providers (AE); 7. Navigating the Health System(NSH); 8. Ability to Find Good Health Information (FHI) 9. Understanding Health Information Well Enough to Know What to Do (UHI) These scales are evaluated with a score that can range from one to five, and the answers range from “always difficult” to “always easy” ^( 7 , 10 )^.

The HLQ does not provide an overall score, but scores for each of the scales separately. The score indicates each person’s strengths and needs concerning their HL ^( 7 , 10 )^. It is calculated by adding up each item on the scale and dividing this value by the number of items on the scale, with the value presented as the average score. The study obtained prior consent from the authors to use the instrument due to copyright (hl-info@swin.edu.au).

## Data collection

The instruments were applied at the bedside, face to face, using a cell phone or tablet. Every two days, the patient’s electronic medical record was accessed to check for new admissions. The interviewer then went to the patient’s bedside and invited them to take part at that moment and, if there was any impossibility, the assistant left the room and returned later. People answered the questionnaire in the absence of a caregiver or family member, guaranteeing privacy and ethical aspects. Four research assistants, nursing and medical students, took part in the data collection. They had previously been trained by the project coordinator, i.e. the last author, by reading and monitoring the initial data collection, as well as following it up until it was completed. The interviews lasted approximately 30 minutes.

## Data processing and analysis

The results were presented using descriptive statistics and measures of central tendency and variability. The symmetry of the continuous distributions was studied using the Kolmogorov-Smirnov test. Cronbach’s alpha was calculated to check the reliability of the instrument. The t-student test was used to compare the continuous variables between the two independent groups. When the comparison of scores involved three or more independent groups, the Analysis of Variance (One Way) - Bonferroni *post hoc* was used. The linear relationship between ages and scale scores was analyzed using Pearson’s correlation coefficient. The statistical treatment was carried out using the Statistical Package for the Social Sciences (SPSS) statistical program, version 25.0 (SPSS Inc., Chicago, IL, USA, 2018) for Windows, and a 5% significance level was adopted for the statistical decision criteria.

## Ethical aspects

The study respected the ethical aspects of Resolution 466/2012. The research was approved on February 28, 2023, by the Research Ethics Committee (REC), under No. 66693823.7.0000.5350 and Opinion No. 5.915.197.

## Results

Of the 166 patients with NCDs, the most prevalent chronic diseases were cardiovascular (153; 92.2%), metabolic (40; 24.1%), neurological (41; 24.7%), and neoplasms (38; 22.9%). There was a predominance of female patients (87; 52.4%), with a mean age of 69.3 ± 14.3 years, white (159; 95.8%), and income of up to five minimum wages (121; 73%). The majority (158; 95.8%) reported knowing how to read and write. Regarding education, (92;55.4%) patients did not study or had completed elementary school, (40; 24.1%) high school, and (34; 20.5%) higher education. Regarding their participation in the Preventive Medicine program, the majority reported not participating (117; 70.5%). Regarding the length of time with chronic disease, the majority said they had the disease for more than 14 years (70; 42.2%) (Table 1).

**Table 1 -:** Sociodemographic and clinical characterization of people living with chronic non-communicable diseases. Ijuí, RS, Brazil, 2023

**Variables**	**Total sample (n=166)**	
	**n**	**%**
**Sex**		
Female	87	52.4
Male	79	47.6
**Age group (years)**		
Up to 49	17	10.2
50 to 69	52	31.3
Over 69	97	58.4
**Age (years)**		
Mean±SD*	69.3±14.3	
**Color**		
White	159	95.8
Non-white	7	4.2
**Marital status**		
Living in a stable union	107	64.5
Not in union	59	35.5
**Monthly family income**		
Up to 2 MW ^†^	30	18.1
3 to 5 MW ^†^	65	39.2
6 to 10 MW ^†^	30	18.1
Above 10 MW ^†^	15	9.0
No answer	26	15.7
**Can read**		
No	8	4.2
Yes	158	95.2
**Can write**		
No	7	4.3
Yes	159	95.7
**Schooling**		
No schooling/Elementary school	92	55.4
High School	40	24.1
Higher Education Complete	34	20.5
**Preventive Medicine Program**		
No	117	70.5
Yes	49	29.5
**Time with CNCD** ^‡^		
Up to 1 year	19	11.4
1 to 5 years	34	20.5
5 to 14 years	43	25.9
Over 14 years	70	42.2

*Mean±SD = Standard deviation; ^†^SW = Minimum wage - R$1,302.00 (Brazil, 2023); ^‡^CNCD = Chronic non-communicable diseases

Table 2 shows the HLQ averages and the instrument’s internal consistency.

**Table 2 -:** Measures of central tendency, variability, and internal consistency of the HLQ* scales. Ijuí, RS, Brazil, 2023

HLQ scales*	**Mean**	**Standard deviation**	αC ^†^
**Part 1- Score 1-4**			
1. Feeling understood and supported by healthcare providers	3.3	0.5	0.778
2. Having sufficient information to manage one’s health	2.8	0.6	0.821
3. Active Health Care	2.9	0.5	0.658
4. Social Support for Health	3.5	0.4	0.741
5. Appraisal of Health Information	2.5	0.7	0.813
**Part 2- Score from 1-5**			
6. Ability to actively engage with healthcare providers	3.9	0.9	0.860
7. Navigating the Health System	3.6	1.0	0.870
8. Ability to Find Good Health Information	3.1	1.0	0.825
9. Understand Health Information Well Enough to Know What to Do	3.2	0.9	0.787

*HLQ = Health Literacy Questionnaire; ^†^αC = Cronbach›s Coefficient Alpha

When evaluating the LS scores, it was identified that the scales that had the best averages, considered strengths, were: Ability to Actively Interact with Health Professionals, Navigating the Health System and Social Support for Health, therefore considered strengths. And the scales Assessment of Health Information, Sufficient Information to Take Care of Health and Ability to Find Good Health Information, with lower averages, considered weaknesses (Table 2).

When comparing the age groups with the scales Understanding and Support from Health Professionals (HPS); Sufficient Information for Health Care (HSI); Active Health Care (AMH); Social Support for Health (SS), it was found that there were no statistically significant differences. A weak but significant correlation was found in the Social Support for Health (SS) scale with the older age of the study participants (r=0.153; p=0.049) (Table 3).

**Table 3 -:** Mean and standard deviation for the HPS*, HSI^†^, AMH^‡^, and SS^§^ scales of the HLQ^||^ scale, according to specific characteristics of the sample profile. Ijuí, RS, Brazil, 2023

**Variables**	**N**	**HLQ** ^||^			
		HPS*	**HSI** ^†^	**AMH** ^‡^	**SS** ^§^
		**Mean±SD** ^¶^	**Mean±SD** ^¶^	**Mean±SD** ^¶^	**Mean±SD** ^¶^
**Age group (years)**					
Up to 49	17	3.3 ± 0.4	2.7 ± 0.6	2.9 ± 0.4	3.5 ± 0.5
50 to 69	52	3.3 ± 0.5	2.8 ± 0.6	3.0 ± 0.5	3.5 ± 0.5
Over 69	97	3.4 ± 0.5	2.8 ± 0.7	2.9 ± 0.5	3.6 ±0.4
P **		0.793	0.689	0.227	0.381
**Age (years) - *r* (p)** ^††^		0.094 (p=0.226)	0.087 (p=0.265)	-0.048 (p=0.539)	0.153 (p=0.049)
**Schooling**					
Did not study/ Elementary school	92	3.3 ± 0.5	2.8 ± 0.7	2.9 ± 0.5	3.6a ^‡‡^ ± 0.4
Elementary	40	3.4 ± 0.5	2.8 ± 0.6	3.0 ± 0.4	3.5ab ± 0.4
High school	34	3.4 ± 0.5	2.7 ± 0.6	2.9 ± 0.5	3.4b ± 0.5
p **		0.898	0.793	0.892	0.009
**Can read**					
Yes	158	3.3 ± 0.5	2.8 ± 0.6	2.9 ± 0.5	3.5 ± 0.4
No	8	3.4 ± 0.7	2.8 ± 1.1	2.9 ± 0.6	3.7 ± 0.4
p ^§§^		0.564	0.943	0.668	0.175
**Can write**					
Yes	159	3.3 ± 0.5	2.8 ± 0.6	2.9 ± 0.5	3.5 ± 0.4
No	7	3.4 ± 0.7	2.8 ± 1.2	3.0 ± 0.5	3.7 ± 0.4
p ^§§^		0.777	0.843	0.790	0.311
**Preventive Medicine Program**					
Yes	49	3.4 ± 0.4	3.0 ^||||^ ± 0.6	3.0 ± 0.5	3.6 ± 0.4
No	117	3.3 ± 0.5	2.7 ± 0.6	2.9 ± 0.5	3.5 ± 0.4
p ^§§^		0.292	0.015	0.539	0.092
**Time since CNCD** ^⁋⁋^ **diagnosis (years)**					
Up to 1 year	19	3.2 ± 0.4	2.6 ± 0.4	2.8 ± 0.5	3.4 ± 0.4
1 a 5	34	3.2 ± 0.5	2.7 ± 0.7	3.0 ± 0.5	3.5 ± 0.4
5 a 14	43	3.4 a ^||||^ ± 0.5	3.0 ± 0.6	3.0 ± 0.6	3.5 ± 0.4
Over 14	70	3.4 a ^||||^ ± 0.5	2.8 ± 0.7	2.9 ± 0.4	3.5 ± 0.5
p ^††^		0.044	0.106	0.410	0.834

*HPS = Feeling Understood and Supported by Healthcare Providers; ^†^HSI = Having Sufficient Information to Manage One’s Health; ^‡^AMH = Actively managing one’s health; ^§^SS = Social Support for Health; ^||^HLQ = Health Literacy Questionnaire; ^¶^Mean± SD = Standard Deviation; **Analysis of Variance (One Way) - Bonferroni *post hoc*; “a” significantly higher mean score, compared to the other means of the specific scale; “b” intermediate average score, which differs significantly from the highest average, as well as from the lowest average; ‘c’ significantly lower average, compared to the other averages of the specific scale; ^††^r(p) = Pearson’s correlation; ^‡‡^Statistically significant difference at 1%, estimated by the analysis of variance test (One Way) - Bonferroni *post hoc*; ^§§^Student’s t-test for independent groups; ^||||^Statistically significant difference at 5%; ^⁋⁋^CNCD = Chronic non-communicable diseases

When comparing the age groups with the scales Understanding and Support from (HPS); Sufficient Information for Health Care(HIS); Active Health Care (AMH); Social Support for Health Support (SS), age groups were compared in terms of the scales in part 1, with no statistically significant differences (Table 3).

When comparing the same scale, it was possible to identify higher scores in the group with up to elementary school education (p=0.009). There was no significant difference in scores between the two higher levels of education. The results observed in the other scales showed that the differences between the scores of the different levels of education were not representative. No significant differences were identified in the characteristics of being able to read or write (Table 3).

The participants were compared to those not taking part in the Preventive Medicine program, and a statistically significant difference was found between those investigated who were part of the Preventive Medicine program, on the scale, Sufficient Health Care Information (HSI) (p=0.015). As for the other scales, there were no representative results (Table 3).

When comparing patients concerning the length of time they had CNCD, a statistically significant difference was identified in patients diagnosed between five and 14 years and over 14 years, with higher average scores on the “Feeling understood and supported by healthcare providers” (HPS) scale (p=0.044) (Table 4).

Table 5 shows a statistically significant difference between the age groups for the Appraisal of Health Information (CA) scales (p<0.001). A higher average score was found in the age groups up to 49 years and 50 to 69 years. According to the definition of the CA scale, high means that the person can identify good information and reliable sources, and resolve conflicting information alone or with the help of other people, and this ability was present in those investigated in the age groups up to 49 years and 50 to 69 years.

**Table 4 -:** Mean and standard deviation for the CA*, AE^†^, and NHS^‡^ scales of the HLQ^§^ scale, according to sociodemographic characteristics. Ijuí, RS, Brazil, 2023

**Variables**	**N**	**HLQ** ^§^		
		CA*	**AE** ^†^	**NHS** ^‡^
		**Mean ± SD** ^||^	**Mean ± SD** ^||^	**Mean ± SD** ^||^
**Age group (years)**				
Up to 49	17	2.8a ^¶^ ± 0.6	3.8 ± 0.9	3.5 ± 1.0
50 to 69	52	2.8 a ^¶^ ± 0.6	3.9 ± 0.8	3.9 a** ± 0.8
Over 69	97	2.3 ± 0.7	3.8 ± 0.9	3.4 ± 1.0
p ^††^		<0.001	0.697	0.018
**Age (years) - r(p)** ^‡‡^		-0.301 (p<0.001)	-0.049 (0.529)	-0.133 (p=008)
**Schooling**				
No schooling/Elementary school	92	2.2 ± 0.7	3.8 ± 0.9	3.4 ± 1.0
High school	40	2.7 b ^¶^ ± 0.6	3.9 ± 0.8	3.7 ± 0.9
Higher education	34	3.0 a ± 0.6	4.0 ± 0.9	3.8 ± 0.8
p ^††^		<0.001	0.667	0.094
**Can read**				
Yes	158	2.5** ± 0.7	3.9 ± 0.9	3.5 ± 1,0
No	8	1.9 ± 0.8	4.1 ± 0.7	3.7 ± 1.2
P ^§§^		0.010	0.532	0.594
**Can write**				
Yes	159	2.5** ± 0.7	3.8 ± 0.9	3.5 ± 1.0
No	7	1.9 ± 0.9	4.2 ± 0.6	4.1 ± 0.7
p ^§§^		0.033	0.296	0.129

*CA = Appraisal of Health Information; ^†^AE = Ability to Actively Engage with Healthcare Providers; ^‡^NHS = Navigating the Health System; ^§^HLQ = Health Literacy Questionnaire; ^||^Mean ± SD = Standard Deviation; ^¶^Statistically significant difference at 1%, estimated by the Analysis of Variance (One Way) - *post hoc* Bonferroni test; **Statistically significant difference at 5%; ^††^Analysis of Variance (One Way) - *post hoc* Bonferroni; “a” Significantly higher average score, compared to the other averages of the specific scale; ‘b’ Intermediate average score, which differs significantly from the highest average, as well as from the lowest average; ‘c’ Significantly lower average, compared to the other averages of the specific scale; ^‡‡^r(p)Pearson’s correlation; ^§§^Student’s t-test for independent groups

**Table 5 -:** Mean and standard deviation for the FHI* and UHI^†^ scales of the HLQ^‡^ scale, according to sociodemographic characteristics. Ijuí, RS, Brazil, 2023

**Variables**	**N**	**HLQ** ^‡^	
		FHI*	**UHI** ^†^
		**Mean ± SD** ^§^	**Mean ± SD** ^§^
**Age group (years)**			
Up to 49	17	3.5 a ^¶^ **± 1** .1	3.5 a ^¶^ **± 1** .1
50 a 69	52	3.4 ^¶^ **± 0** .8	**3.5** ^¶^ **± 0** .7
Over 69	97	2.9 **± 1** .0	3.0 **± 0** .9
p ^||^		0.002	0.001
Age (years) - r(p)**		-0.334 (p<0.001)	-0.301 (p<0.001)
Schooling			
No schooling/Elementary school	92	2.8 **± 0** .9	3.0 **± 0** .9
High school	40	3.5 a ^¶^ **± 0** .9	3.3 b ^¶^ **± 0** .8
Higher education	34	3.8 b **± 0** .8	3.6 a ^¶^ **± 1** .0
p ^||^		**<** 0.001	0.002
**Can read**			
Yes	158	3.2 ^††^ **± 1** .0	3.2 ^††^ **± 0** .9
No	8	2.4 **± 0** .7	2.0 **± 0** .5
p ^‡‡^		0.008	**<** 0.001
**Can write**			
Yes	159	3.2	3.2 ^††^
No	7	2.5	2.0
p ^‡‡^		0.071	0.001

*FHI = Ability to Find Good Health Information; ^†^UHI = Understanding Health Information Well Enough to Know What to Do; ^‡^HLQ = Health Literacy Questionnaire; ^§^Mean±SD = Standard Deviation; ^||^Analysis of Variance (One Way) - *post hoc*; Results with a statistically significant difference have the notation: “a” significantly higher average score, compared to the other averages of the specific scale; ‘b’ intermediate average score, which differs significantly from the highest average, as well as from the lowest average; ‘c’ significantly lower average, compared to the other averages of the specific scale; ^¶^Statistically significant difference at 1%, estimated by the Analysis of Variance (One Way) - *post hoc* Bonferroni test; **r(p)= Pearson’s Correlation; ^††^Statistically significant difference at 5%; ^‡‡^Student’s t-test for independent groups

The age of those investigated was also compared to the scales that guided the comparisons in Table 4, and a statistically significant and negative correlation was detected in the CA scale (r = -0.301; p<0.001), which pointed to the high scores in the Health Information Evaluation scale, which must be correlated to the lower ages observed in the sample.

The Navigating the Healthcare System (NHS) scale (p=0.018) also stood out with a significant result, with the average score in the 50-69 age group being significantly higher when compared to the others. There was no significant result for the Ability to Actively Engage with Healthcare Providers (AE) scale. The results of the correlations between age and the AE and NHS scales were not representative. When considering the comparison of schooling concerning the scales, a statistically significant difference was identified with the CA scale (p<0.0001). Patients with high school and higher education had significantly higher mean scores when compared to those with lower levels. There were no significant differences in the AE and NHS scales.

Concerning being able to read, the significant difference was only seen on the CA scale (2.5±0.7 vs. 1.9± 0.9 p=0.010), where the group that declared they could read had a significantly higher average score compared to the others.

A similar result was observed concerning the variable knowing how to write when comparing the scales. The only significant difference was in the CA scale (p=0.032), where those who reported knowing how to write had a significantly higher average score.

When considering the analyses involving age group comparisons, in the Ability to Find Good Health Information (FHI) scale, there was a statistically significant difference (p=0.002), and indicated that the average scores in the age groups up to 49 years and 50 to 59 years were significantly higher. There was a similar result when comparing the age group with the scale: Understanding Health Information Well Enough to Know What to Do (UHI) (p=0.001).

According to the scale’s definition, there is evidence that the age groups up to 49 and 50 to 69 showed greater ability to explore information. The two age groups also showed a greater ability to understand written health information.

When correlating the scales with age, statistically significant and negative correlations were identified, both between age and the FHI scale (r = -0.334; p<0.001), and on the UHI scale data (r = -0.301; p<0.001). There is evidence that the highest age groups were correlated with the lowest scores.

Regarding comparisons on the question of knowing how to read, some statistically significant differences were detected in the scores on the FHI and UHI scales. It was found that the average score was significantly higher among those who reported being able to read FHI (p=0.010). A similar result occurred when comparing the variables “Can read”. When comparisons were made with the UHI scale on the question of knowing how to write (p=0.010), the statistically significant difference was exclusively on this scale, with a higher average score in the group who reported knowing how to write.

## Discussion

This is the first Brazilian study to evaluate the HL of patients living with CNCDs and to verify its relationship with sociodemographic variables (age, schooling, being able to read and write, and whether it is part of Preventive Medicine) and clinical variables (time since CNCD diagnosis), using bivariate analysis. The highest scores were found in the Social Support for Health and Ability to Actively Interact with Health Professionals scales, both results being similar to those of another international study of people living in a rural fishing community in northern Egypt ^( 13 )^.

People with CVD predominated, similar to a study that evaluated HL in Brazil with people affected by CNCDs, in which CVD predominated with 230 people (29%) and females (67.5%) ^( 10 )^. Other current studies have also reaffirmed these results ^( 1 , 6 )^. A reduction in HL was observed with increasing age and lower schooling. HL plays a significant role in the management of CNCDs, which are considered complex and become a significant and long-term challenge for people and the health system ^( 4 , 6 )^.

The lowest scores were found in the “Appraisal of Health Information” and “Ability to Find Good Health Information” scales, which corroborates other Brazilian studies that used the same instrument with people living with HIV and chronic kidney disease ^( 12 , 14 )^.

In this study, patients with advanced ages and low levels of education performed best on the “Social Support for Health” scale, a result similar to that found in other studies ^( 15 )^. In addition, quality social support promotes better health outcomes ^( 16 )^ and self-management ^( 17 )^. To this end, providing education and support to family members and caregivers involved in this context can be a useful strategy for health services, since other studies ^( 18 - 19 )^ suggest that caregivers of people with CNCD need care information, and the principles of HL are crucial in the daily lives of caregivers, professionals and managers for better health outcomes and decisions ^( 11 )^.

A study carried out in Australia showed that patients with advanced ages and lower levels of education need and/or receive more Social Support for Health, which favors HL in this population ^( 20 )^. Another study in Brazil showed similar results to this one ^( 14 , 21 )^.

It is essential that health professionals assess patients’ HL levels and adapt information and support to HL skills according to the context of their patients, in a unique way, as this favors the management of their chronic health condition throughout their lives ^( 9 )^. Strategies based on assertive communication, understandable information, and clear access to services favor people’s HL ^( 7 , 22 )^.

Data from this study showed that people who were part of a Preventive Medicine program had a better average score on the “Having Sufficient Information to Manage My Health” scale. In other words, they felt confident that they had the information they needed to live together, manage their conditions, and make decisions about their health ^( 8 )^, unlike other studies which showed lower averages ^( 12 - 13 )^, which highlights the importance of programs based on HL principles ^( 3 )^.

It is important to have an established relationship with at least one health professional who knows them well and whom they can trust to give them advice and useful information, to help them understand the guidelines and make decisions about their health ^( 8 )^. Strengthening the bond facilitates continuity of care and provides an opportunity to develop strategies uniquely. It is considered strategic that the transmission of information by health professionals is clear and in accessible language, to favor understanding and encourage self-management ^( 23 )^, given that health services are the main gateway and are considered a safe source of information for users.

It was found that the participants in this study showed weaknesses in the “Having sufficient information to manage my health” and “Appraisal of Health Information” scales. They had gaps in their health knowledge, were unable to understand the information they received, and believed they did not have the necessary information for self-management, which corroborated another study ^( 12 )^ that identified a low/insufficient degree of adherence to drug treatment in people with HIV, due to weaknesses in these scales.

These results have important implications and reaffirm the need for, advances in, care for people with CNCDs, in terms of assertive communication between patients and professionals in a unique way, as has been shown in other studies with similar results ^( 11 , 24 - 25 )^. Access to information is not the main problem, but rather its quality, since people need to understand the guidelines clearly to self-manage. To this end, the availability of educational materials about the disease and treatment in accessible language and from a safe source are resources that can be adopted ^( 7 , 23 )^.

Assertive communication can be employed through the use of teach-back, which favors the understanding of information ^( 26 )^. These strategies provide support for people to increase their knowledge, capacity for shared decision-making, engagement, and self-management ^( 26 - 27 )^.

This study found that younger people with a higher level of education had HL potential to identify good information in reliable sources, as well as to understand conflicting information on their own or with the help of others. The level of education is relevant since people with a higher level of education can gain better access to information about health care and the treatment of CNCDs ^( 28 )^.

Although this has an influence, it is not always considered a determining characteristic, since people with high levels of education may have insufficient HL, this is unique and transcends knowing how to read or write, considering decision-making skills, health promotion, access, and navigation in the health system ^( 7 )^.

The “Actively Managing One’s Health” scale also showed weaknesses for HL. It was related to taking responsibility for one’s health and being proactively involved in making one’s own decisions. This may be associated with age and low schooling, as advanced age can reduce the ability to perform cognitive tasks that require information processing. Older people seem to have more difficulty completing tasks that require them to reason or draw conclusions from the information presented to them ^( 29 )^.

In this sense, some authors have pointed out that social support should be a concern for health professionals, especially nursing professionals, in order to improve educational practices that can provide qualified listening, with clarification on care, and even considering emotional issues ^( 30 )^, with a view to a comprehensive approach to the elderly and those with greater vulnerability.

When considering the scales: “Ability to Find Good Health Information” and “Understanding Health Information Well Enough to Know What to Do”, it was identified that, up to the age of 69, people had HL potential. People need to be able to explore, understand, and use different sources of information and be able to write appropriately on forms when necessary.

The study made it possible to identify aspects that need to be improved in care, to support the development of strategies and encourage HL. The study made it possible to see that investment in health policies, both public and private, based on HL strategies is crucial, as is the need to support managers, health professionals, and engaged users so that change can take place.

In addition, the results can contribute to other studies published and underway at a universal level, to facilitate reflection on HL in health management practices in Brazil and other countries.

This study has some limitations, such as the type of study chosen, quantitative and cross-sectional, which does not allow establishing a causal relationship between the variables, as well as its performance in a single general hospital. Therefore, caution should be taken when generalizing the results. Despite the limitations, this study brings innovations regarding the importance of considering HL for the qualification of health care in NCDs. The results can provide support for the deepening of future research investigating the causal relationship between HL and sociodemographic and clinical variables in patients hospitalized with NCDs.

## Conclusion

The patients showed potential in HL, on the scales “Social Support for Health” (3.5±0.4), “Ability to Actively Engage with Healthcare Providers” (3.9±0.9), and “Navigating the Health System” (3.6±1.0); on the other hand, weaknesses in the evaluation of services (2.5±1.0) and in the “Ability to Find Good Health Information” (3.1±1.0).

There was a significant association between HL and sociodemographic variables such as age, schooling, and being able to read and write. Patients aged up to 49 and 50 to 59 had lower mean scores on the “Social Support for Health” scale, while those with less schooling had higher mean scores. The younger age groups showed the best HL results in the following scales: “Assessing Health Services”, “Ability to Find Good Health Information”, and “Understanding Health Information Well Enough to Know What to Do”.

Younger patients with more schooling and reading and writing skills had higher scores on scales related to evaluating and understanding health information. On the other hand, older individuals with less schooling showed lower scores, indicating weaknesses in understanding health information. However, it was identified that the “Social Support for Health” scale showed potential and could favor HL in the evaluation and use of health services.

People who were part of the Preventive Medicine program had higher mean scores on the “Sufficient Information to Take Care of One’s Health” scale. People with more CNCD reported that they were better understood and supported by health professionals.

Given the above, health professionals must reflect on and uniquely implement HL strategies for people with older ages and lower levels of education, so that they can benefit from decision-making about their health and well-being.
