# Supplementary material for: Sociodemographic and clinical characteristics associated with health literacy in people hospitalized for chronic diseases
Source: Rev Lat Am Enfermagem. 2025 May 2;33:e4494. doi: 10.1590/1518-8345.7395.4494 (PMC12052309; doi:10.1590/1518-8345.7395.4494)
Supplement: Translation

# Translation: Características sociodemográficas e clínicas associadas ao letramento em saúde de pessoas hospitalizadas por doenças crônicas*

**DOI:** 10.1590/1518-8345.7395.4495

**Authors:** Francini de Oliveira Rodrigues, Eliana Elisa Rehfeld Gheno, Letícia Y Castro, Arnaldo Nogaro, Amanda Caroline Mélo da Rosa, Adriane Cristina Bernat Kolankiewicz

**Affiliations:** 1 Universidade Regional do Noroeste do Estado do Rio Grande do Sul, Ijuí, RS, Brasil; 2 Universidade do Alto Uruguai e das Missões, Campus Erechim, RS, Brasil; 3 Bolsista do Conselho Nacional de Desenvolvimento Científico e Tecnológico (CNPq), Brasil

**Keywords:** Letramento em Saúde, Doenças Não Transmissíveis, Hospitalização, Doenças Crônicas, Acesso à Informação, Gestão em Saúde

## Abstract

identificar a associação do letramento em saúde com as variáveis sociodemográficas e clínicas de pacientes hospitalizados com doenças crônicas.

estudo transversal, realizado com pacientes com doenças crônicas, internadas em um hospital geral. Utilizaram-se um questionário sociodemográfico/clínico e o *Health Literacy Questionnaire,* composto por nove escalas, mensuradas por escores, com a análise dos dados descritiva e inferencial.

o Suporte Social Para a Saúde foi associado ao nível de escolaridade fundamental (p=0,009). Compreensão e Apoio dos Profissionais de Saúde foram relacionados ao tempo de doença crônica (p=0,044). A Avaliação das Informações de Saúde mostrou-se associada à faixa etária (p=0,001), saber ler (p=0,010) e escrever (p=0,032). Navegar no Sistema de Saúde também esteve relacionado à faixa etária (p=0,018), assim como a Capacidade de Encontrar Boas Informações de Saúde (p=0,002), e saber ler (p=0,010), e Compreender as Informações de Saúde bem o Suficiente para Saber o que Fazer esteve associada à faixa etária (p=0,001) e saber escrever (p=0,010).

escolaridade, faixa etária, tempo de doença crônica, saber ler e escrever, interferem no letramento em saúde. Isso destaca a necessidade de estratégias personalizadas que levem em consideração essas variáveis para melhorar o letramento em saúde em populações com doenças crônicas hospitalizadas.

## Destaques

**(1)** Primeiro estudo brasileiro que avaliou o letramento em saúde de pessoas com DCNT. **(2)** Potencialidades: capacidade de interação, navegar no sistema e suporte social. **(3)** Fragilidades: avaliação das informações de saúde e capacidade de encontrá-las. **(4)** Idade, escolaridade, saber ler e escrever interferem no letramento em saúde.

## Introdução

As Doenças Crônicas Não Transmissíveis (DCNTs) constituem um problema mundial de saúde pública. São consideradas a principal causa de incapacidade e mortalidade prematura no mundo, e respondem pela morte de 40,8 milhões de pessoas, o equivalente a 74% de todas as mortes ^( 1 )^.

O acometimento pelas DCNTs implica no acesso aos bens e serviços públicos, garantia de direitos, informação, emprego, renda e a possibilidade de fazer escolhas favoráveis à saúde ^( 2 )^. Nesse contexto, o Letramento em Saúde (LS) contribui e pode modificar os fatores negativos à saúde e ajudar a compreender os pontos fortes e frágeis das pessoas e das comunidades. Representa o conhecimento e as competências pessoais que se acumulam por meio de atividades cotidianas, interações sociais e gerações. Mediados por estruturas organizacionais e a disponibilidade de recursos que permitem que as pessoas acessem, compreendam, avaliem e usem as informações e os serviços, de maneira que promovam e que mantenham a boa saúde e o bem-estar ^( 3 )^.

O LS é considerado como o melhor indicador de saúde em relação a muitos determinantes sociais ^( 4 )^. O LS insuficiente resulta no uso inadequado dos serviços e gera resultados negativos para a saúde. Além disso, associa-se às altas taxas de hospitalização, ao aumento da prevalência de doenças, ao menor uso de métodos preventivos e à baixa adesão aos tratamentos. Em contrapartida, quando adequado, melhora o autogerenciamento do cuidado, da Qualidade de Vida (QV) e, consequentemente, reduz as reinternações hospitalares, e a sobrecarga e os custos ^( 5 )^.

Nesse contexto, os pacientes com agravamento de sua saúde por DCNTs frequentemente requerem a internação hospitalar, devido à sua complexidade clínica. E os profissionais de saúde que os assistem, em especial a Enfermagem, precisam contribuir no preparo para a alta de modo seguro e de forma que dê continuidade ao cuidado. Para tanto, faz-se necessária a construção de habilidades, considerando o nível de LS, no intuito de facilitar o manejo, o empoderamento, o entendimento do seu autocuidado, e evitar, consequentemente, os eventos adversos e as reinternações ^( 6 )^.

Alguns estudos têm-se limitado a fazer a avaliação das habilidades cognitivas de leitura e numeramento, sem considerar os demais aspectos do LS, que permitem que os indivíduos e a sociedade sejam responsáveis por suas condições de saúde ^( 7 )^. Uma pesquisa com pessoas acometidas com DCNTs, na Etiópia, identificou que os níveis de LS variam de acordo com as características sociodemográficas e clínicas. Nesse sentido, os profissionais de saúde devem avaliar o nível de LS e adaptar as informações e o apoio às competências de LS conforme o contexto dos pacientes, o que favorece a gestão da sua condição crônica durante a vida ^( 8 )^.

No contexto internacional, outros estudos têm identificado a relação do LS com a caracterização sociodemográfica ^( 9 )^, porém não com as condições clínicas. No Brasil, foram realizados estudos de avaliação multidimensional do LS em diferentes populações, como os profissionais de saúde, usuários em geral, cuidadores domiciliares e pessoas com o Vírus da Imunodeficiência Humana (HIV) ^( 10 - 12 )^. No Brasil, o LS é pouco explorado no contexto das práticas e da gestão em saúde, inexistindo uma padronização de estratégias e planos eficazes para melhor LS e sua efetividade. Sobretudo, que possam ser aplicados durante o período de internação, pois poucas são as instituições que executam as ações com enfoque nestes, para que possam contribuir para a redução de reinternações hospitalares.

Assim, por meio da identificação das associações do LS com as variáveis sociodemográficas e clínicas, os profissionais de Enfermagem e as instituições de saúde poderão reconhecer as fragilidades do LS e desenvolver as estratégias eficazes para as pessoas com doenças crônicas, a fim de reduzir as reinternações. Reconhece-se a escassez de estudos nacionais e internacionais que relacionem o LS com a caracterização sociodemográfica e clínica, na perspectiva de pessoas que vivem com DCNTs. Diante desse contexto, e da lacuna do conhecimento identificado, é que se justifica a realização deste estudo.

Assim, este estudo objetiva identificar a associação do LS com as variáveis sociodemográficas e clínicas de pacientes hospitalizados com doenças crônicas.

## Método

### Tipo de estudo

Trata-se de um estudo quantitativo e transversal baseado nas diretrizes do *Strengthening the Reporting of Observational Studies in Epidemiology* (STROBE). Salienta-se que o LS no contexto hospitalar será analisado na perspectiva de pacientes com doenças crônicas, a partir das variáveis sociodemográficas e clínicas.

### Contexto e coleta de dados

O estudo foi realizado em um hospital geral com 76 leitos de unidades de internação clínica, na cidade de Ijuí, Rio Grande do Sul (RS), Brasil. As unidades são compostas por 18 leitos privativos e 58 leitos não privativos duplos. A instituição oferece um programa de “Medicina Preventiva em Atenção Integral à Saúde”, que acompanha os pacientes por meio de atividades de atenção integral e preparo para a alta hospitalar.

### Período

A coleta de dados foi realizada entre os meses de abril e outubro de 2023.

### Participantes

Participaram da pesquisa 166 pessoas hospitalizadas com DCNTs.

### Critérios de seleção

Critérios de inclusão: os pacientes maiores de 18 anos, com diagnóstico de DCNTs registrado em prontuário de saúde, internados por pelo menos 24 horas. Foram excluídos parturientes, pacientes cirúrgicos, institucionalizados após a alta, e pessoas com atividade cognitiva não preservada, que foi avaliada pela primeira autora, enfermeira, via registros no prontuário de saúde e presencialmente à beira do leito, a partir da avaliação do nível de consciência do paciente, bem como do conteúdo.

### Definição da amostra

A população do estudo foi constituída pelos pacientes internados com DCNTs na instituição, no período de abril a agosto de 2023, o que alcançou um tamanho populacional, de aproximadamente 370 pacientes, o que caracteriza uma população finita. Como estimativa para a base de cálculo sobre o tamanho amostral, considerou-se o principal fator de estudo, a escala *Health Literacy Questionnaire* (HLQ). Para o total da escala foi assumido escore médio de 3,0 (±1,0), que define o ponto médio.

Com base nessa estimativa e ao assumir o nível de significância de 5% (α=0,05), uma margem de erro de 7,5% sobre a média a ser estimada, que equivale a uma variação de 0,2 unidades, o tamanho mínimo de amostra foi definido em 159 pacientes. Ao aplicar o fator de correção para as populações finitas, o tamanho mínimo necessário, para este estudo foi estimado em 112 investigados. Dessa forma, como alcançaram 166 investigados, foi satisfeito um tamanho amostral que validasse a representatividade amostral.

Diariamente os prontuários eletrônicos eram analisados, para a identificação do perfil clínico do paciente e o tempo de internação, e foram convidados durante o período de coleta de dados. Dos prontuários analisados, 192 pacientes foram convidados a participarem da pesquisa e, destes, 26 recusaram. Assim, a amostra foi constituída por 166 pacientes internados com DCNTs.

### Variáveis do estudo

Foram consideradas as variáveis sociodemográficas idade, sexo, renda, escolaridade, raça, estado civil, fazer parte do programa da Medicina Preventiva, saber ler e escrever. E as variáveis clínicas como: condições de saúde, tempo de diagnóstico, ter uma ou mais doenças crônicas. Para o LS utilizaram-se as escalas do questionário HLQ.

## Instrumentos utilizados para a coleta das informações

No prontuário de saúde foram obtidas as informações a respeito das variáveis clinicas dos pacientes. Para as variáveis sociodemográficas e LS utilizaram-se um questionário sociodemográfico e o HLQ, respectivamente, à beira do leito. O HLQ é um instrumento multidimensional para mensurar o LS ^( 7 )^, validado para uso no Brasil ^( 10 )^.

O HLQ engloba 44 itens e é subdividido em duas partes. Na parte 1, tem-se as escalas: 1. Compreensão e Apoio dos Profissionais de Saúde (HPS); 2. Informações Suficientes Para Cuidar da Saúde (HSI); 3. Cuidado Ativo da Saúde (AMH); 4. Suporte Social Para a Saúde (SS); 5. Avaliação das Informações de Saúde (CA). Nestas escalas o escore para o cálculo da média é de um a quatro, com respostas variando entre “discordo totalmente” e “concordo totalmente” ^( 7 , 10 )^.

Na parte 2, tem-se as escalas: 6. Capacidade de Interagir Ativamente com os Profissionais de Saúde (AE); 7. Navegar no Sistema de Saúde (NHS); 8. Capacidade de Encontrar Boas Informações de Saúde (FHI); 9. Compreender as Informações de Saúde bem o Suficiente para Saber o que Fazer (UHI). Estas escalas são avaliadas com escore que pode variar de um a cinco, e as respostas vão de “sempre difícil” a “sempre fácil” ^( 7 , 10 )^. O HLQ não fornece pontuação global e, sim, escores para cada uma das escalas separadamente. A pontuação indica pontos fortes e as necessidades de cada pessoa em relação ao seu LS ^( 7 , 10 )^. O cálculo é realizado pela soma de cada item das escalas, e esse valor é dividido pelo número de itens da escala, sendo o valor apresentado como a média da pontuação. O estudo obteve anuência prévia dos autores para uso do instrumento em atendimento aos direitos autorais para sua utilização (hl-info@swin.edu.au).

## Coleta de dados

Os instrumentos foram aplicados à beira do leito, face a face, com o auxílio do telefone celular ou *tablet*. A cada dois dias, era acessado o prontuário eletrônico do paciente para verificar as novas internações. Na sequência, o entrevistador se dirigia até o leito do paciente e o convidava para participar do estudo e, caso houvesse alguma impossibilidade, o auxiliar se retirava do quarto e retornava posteriormente. As pessoas responderam o questionário na ausência de cuidador ou familiar, garantindo a privacidade e os aspectos éticos. Participaram da coleta de dados quatro auxiliares de pesquisa, estudantes de Enfermagem e Medicina, previamente capacitados pela coordenadora do projeto, ou seja, a última autora, por meio da leitura e do acompanhamento na coleta de dados inicial, bem como o seguimento, até encerrá-la. As entrevistas tiveram tempo aproximado de 30 minutos.

## Tratamento e análise dos dados

Os resultados foram apresentados por meio da estatística descritiva, pelas medidas de tendência central e de variabilidade. A simetria das distribuições contínuas foi estudada pelo teste de Kolmogorov-Smirnov. Para verificar a confiabilidade do instrumento calculou-se o alfa de Cronbach. Na comparação das variáveis contínuas entre os dois grupos independentes, foi utilizado o teste t-Student. Quando a comparação dos escores envolveu três ou mais grupos independentes, empregou-se a Análise de Variância (*One Way*) – *post hoc* Bonferroni. A relação de linearidade que envolveu as idades, os escores das escalas foi analisado por meio do coeficiente de correlação de Pearson. O tratamento estatístico foi realizado com auxílio do programa estatístico *Statistical Package for the Social Sciences* (SPSS), versão 25.0 (SPSS Inc., Chicago, IL, USA, 2018) para *Windows*, e para os critérios de decisão estatística foi adotado o nível de significância de 5%.

## Aspectos éticos

O estudo respeitou os aspectos éticos conforme a Resolução n^o^ 466/2012. A pesquisa foi aprovada em 28 de fevereiro de 2023 pelo Comitê de Ética em Pesquisa (CEP), com nº 66693823.7.0000.5350 e o Parecer nº 5.915.197.

## Resultados

Dos (100%) pacientes com DCNTs, as doenças crônicas mais prevalentes foram 153 (92,2%) cardiovasculares, 40 (24,1%), metabólicas, 41 (24,7%), neurológicas e 38 (22,9%), neoplasias. Houve predomínio de pacientes do sexo feminino (87; 52,4%), com média de idade de 69,3 ±14,3 anos, de cor branca (159; 95,8%), com renda de até cinco salários-mínimos (121; 73%). A maioria (158; 95,8%) referiu saber ler e escrever. Em relação à escolaridade, (92;55,4%) pacientes não estudaram ou tinham Ensino Fundamental, (40; 24,1%), cursaram Ensino Médio e (34; 20,5%), o Ensino Superior completo. Quanto a sua participação no programa de Medicina Preventiva, a maioria referiu que não participava (117; 70,5%). Em relação ao tempo de doença crônica, a maioria referiu que tinha a doença há mais de 14 anos (70; 42,2%) (Tabela 1).

**Tabela 1 -:** Caracterização sociodemográfica e clínica de pessoas com doenças crônicas não transmissíveis. Ijuí, RS, Brasil, 2023

**Variáveis**	**Total da amostra ( n=166)**	
	**n**	**%**
**Sexo**		
Feminino	87	52,4
Masculino	79	47,6
**Faixa etária (anos)**		
Até 49	17	10,2
50 a 69	52	31,3
Superior a 69 anos	97	58,4
**Idade (anos)**		
Média±DP*	69,3±14,3	
**Cor**		
Branca	159	95,8
Não branca	7	4,2
**Estado civil**		
Vive em união estável	107	64,5
Não vive em união	59	35,5
**Renda familiar mensal**		
Até 2 SM ^†^	30	18,1
3 a 5 SM ^†^	65	39,2
6 a 10 SM ^†^	30	18,1
Acima de 10 SM ^†^	15	9,0
Não respondeu	26	15,7
**Sabe ler**		
Não	8	4,2
Sim	158	95,2
**Sabe escrever**		
Não	7	4,3
Sim	159	95,7
**Escolaridade**		
Não estudou/Ensino Fundamental	92	55,4
Ensino Médio	40	24,1
Ensino Superior Completo	34	20,5
**Programa de Medicina Preventiva**		
Não	117	70,5
Sim	49	29,5
**Tempo de DCNT** ^‡^		
Até 1 ano	19	11,4
1 a 5 anos	34	20,5
5 a 14 anos	43	25,9
Acima de 14 anos	70	42,2

*Média±DP = Desvio-padrão; ^†^SM = Salário-mínimo - R$1.302,00 (Brasil, 2023); ^‡^DCNT = Doença crônica não transmissível

Na Tabela 2 são apresentadas as médias do HLQ e a consistência interna do instrumento.

**Tabela 2 -:** Medidas de tendência central, de variabilidade e consistência interna das escalas do HLQ*. Ijuí, RS, Brasil, 2023

Escalas HLQ*	**Média**	**Desvio-padrão**	αC ^†^
**Parte 1- Escore de 1-4**			
1. Compreensão e Apoio dos Profissionais de Saúde	3,3	0,5	0,778
2. Informações Suficientes Para Cuidar da Saúde	2,8	0,6	0,821
3. Cuidado Ativo da Saúde	2,9	0,5	0,658
4. Suporte Social Para a Saúde	3,5	0,4	0,741
5. Avaliação das Informações de Saúde	2,5	0,7	0,813
**Parte 2- Escore de 1-5**			
6. Capacidade de Interagir Ativamente com os Profissionais de Saúde	3,9	0,9	0,860
7. Navegar no Sistema de Saúde	3,6	1,0	0,870
8. Capacidade de Encontrar Boas Informações de Saúde	3,1	1,0	0,825
9. Compreender as Informações de Saúde bem o Suficiente para Saber o que Fazer	3,2	0,9	0,787

*HLQ = *Health Literacy Questionnaire*; ^†^αC = Coeficiente alfa de Cronbach

Ao avaliar os escores do LS identificou-se que as escalas que tiveram melhores médias, consideradas pontos fortes, foram: Capacidade de Interagir Ativamente com os Profissionais de Saúde, Navegar no Sistema de Saúde e Suporte Social Para a Saúde, portanto, consideradas potencialidades. E as escalas Avaliação das Informações de Saúde, Informações Suficientes Para Cuidar da Saúde e Capacidade de Encontrar Boas Informações de Saúde, com menores médias, consideradas fragilidades (Tabela 2).

Ao comparar as faixas etárias com as escalas Compreensão e Apoio dos Profissionais de Saúde (HPS), Informações Suficientes Para Cuidar da Saúde (HSI), Cuidado Ativo da Saúde (AMH) e Suporte Social Para a Saúde (SS), não foram encontradas diferenças estatísticas significativas. Encontrou-se correlação fraca significativa na escala Suporte Social Para a Saúde (SS) para os pacientes com idade mais avançada (r=0,153; p=0,049) (Tabela 3).

**Tabela 3 -:** Média e desvio-padrão para as escalas HPS*, HSI^†^, AMH^‡^ e SS^§^ da escala HLQ^||^, segundo características específicas da amostra. Ijuí, RS, Brasil, 2023

**Variáveis**	n	**HLQ** ^||^			
		HPS*	**HSI** ^†^	**AMH** ^‡^	**SS** ^§^
		**Média±DP** ^¶^	**Média± DP** ^¶^	**Media± DP** ^¶^	**Média± DP** ^¶^
**Faixa etária (anos)**					
Até 49	17	3,3 ± 0,4	2,7 ± 0,6	2,9 ± 0,4	3,5 ± 0,5
50 a 69	52	3,3 ± 0,5	2,8 ± 0,6	3,0 ± 0,5	3,5 ± 0,5
Superior a 69	97	3,4 ± 0,5	2,8 ± 0,7	2,9 ± 0,5	3,6 ±0,4
P **		0,793	0,689	0,227	0,381
**Idade (anos) - *r* (p)** ^††^		0,094 (p=0,226)	0,087 (p=0,265)	-0,048 (p=0,539)	0,153 (p=0,049)
**Escolaridade**					
Não estudou/ Ensino Fundamental	92	3,3 ± 0,5	2,8 ± 0,7	2,9 ± 0,5	3,6a ^‡‡^ ± 0,4
Ensino Médio	40	3,4 ± 0,5	2,8 ± 0,6	3,0 ± 0,4	3,5ab ± 0,4
Ensino Superior	34	3,4 ± 0,5	2,7 ± 0,6	2,9 ± 0,5	3,4b ± 0,5
p **		0,898	0,793	0,892	0,009
**Sabe ler**					
Sim	158	3,3 ± 0,5	2,8 ± 0,6	2,9 ± 0,5	3,5 ± 0,4
Não	8	3,4 ± 0,7	2,8 ± 1,1	2,9 ± 0,6	3,7 ± 0,4
p ^§§^		0,564	0,943	0,668	0,175
**Sabe escrever**					
Sim	159	3,3 ± 0,5	2,8 ± 0,6	2,9 ± 0,5	3,5 ± 0,4
Não	7	3,4 ± 0,7	2,8 ± 1,2	3,0 ± 0,5	3,7 ± 0,4
p ^§§^		0,777	0,843	0,790	0,311
**Programa de Medicina Preventiva**					
Sim	49	3,4 ± 0,4	3,0 ^||||^ ± 0,6	3,0 ± 0,5	3,6 ± 0,4
Não	117	3,3 ± 0,5	2,7 ± 0,6	2,9 ± 0,5	3,5 ± 0,4
p ^§§^		0,292	0,015	0,539	0,092
**Tempo de diagnóstico DCNT** ^⁋⁋^ **(anos)**					
Até 1 ano	19	3,2 ± 0,4	2,6 ± 0,4	2,8 ± 0,5	3,4 ± 0,4
1 a 5	34	3,2 ± 0,5	2,7 ± 0,7	3,0 ± 0,5	3,5 ± 0,4
5 a 14	43	3,4 a ^||||^ ± 0,5	3,0 ± 0,6	3,0 ± 0,6	3,5 ± 0,4
Acima de 14	70	3,4 a ^||||^ ± 0,5	2,8 ± 0,7	2,9 ± 0,4	3,5 ± 0,5
p ^††^		0,044	0,106	0,410	0,834

*HPS = Compreensão e Apoio dos Profissionais de Saúde; ^†^HSI = Informações Suficientes Para Cuidar da Saúde; ^‡^AMH = Cuidado Ativo da Saúde; ^§^SS = Suporte Social Para a Saúde; ^||^HLQ = *Health Literacy Questionnaire*; ^¶^Média± DP = Desvio-padrão; **Análise de Variância (*One Way*) – *post hoc* Bonferroni; “a” escore médio significativamente mais elevado, em relação às demais médias da escala específica; “b” escore médio intermediário, que difere significativamente da média mais elevada, bem como da menor média; “c” média significativamente menor, em comparação às demais médias da escala específica; ^††^r(p) = Correlação de Pearson; ^‡‡^Diferença estatística significativa a 1%, estimada pelo teste de análise de Variância (*One Way*) – *post hoc* Bonferroni; ^§§^Teste t-Student para os grupos independentes; ^||||^Diferença estatística significativa a 5%; ^⁋⁋^DCNT = Doença crônica não transmissível

A comparação das faixas etárias com as escalas Compreensão e Apoio de (HPS), Informação Suficiente para Cuidados de Saúde (HIS), Cuidados de Saúde Ativos (AMH) e Suporte Social Para a Saúde (SS) aconteceu em relação às escalas da parte 1, não havendo diferenças estatisticamente significativas (Tabela 3).

Em relação à escolaridade observaram-se escores maiores nos pacientes com instrução até o Ensino Fundamental (p=0,009). Entre os dois níveis de escolaridade mais elevados, não houve diferença significativa na comparação entre os escores. Os resultados relacionados às demais escalas mostraram que as diferenças entre os escores dos diferentes níveis de escolaridade não foram representativos. Quanto às características saber ler ou saber escrever, não houve diferenças estatísticas significativas (Tabela 3).

Quando comparada a participação ou não dos pacientes no programa de Medicina Preventiva, observou-se diferença estatística significativa entre aqueles que participavam do programa de Medicina Preventiva, na escala Informações Suficientes Para Cuidar da Saúde (HSI) (p=0,015). Quanto às demais escalas, não houve diferenças estatísticas significativas (Tabela 3).

Ao se comparar os pacientes em relação ao tempo de DCNT e as escalas HPS; HSI e AMH, constatou-se diferença estatística significativa para os pacientes com diagnóstico entre cinco e 14 anos e acima de 14 anos de doença, com os escores médios mais elevados na escala Compreensão e Apoio dos Profissionais de Saúde (HPS) (p=0,044) (Tabela 4).

**Tabela 4 -:** Média e desvio-padrão para as escalas CA*, AE^†^ e NHS^‡^ da escala HLQ^§^, segundo as características sociodemográficas. Ijuí, RS, Brasil, 2023

**Variáveis**	n	**HLQ** ^§^		
		CA*	**AE** ^†^	**NHS** ^‡^
		**Média ± DP** ^||^	**Média ± DP** ^||^	**Média ± DP** ^||^
**Faixa etária (anos)**				
Até 49	17	2,8a ^¶^ ± 0,6	3,8 ± 0,9	3,5 ± 1,0
50 a 69	52	2,8 a ^¶^ ± 0,6	3,9 ± 0,8	3,9 a** ± 0,8
Superior a 69	97	2,3 ± 0,7	3,8 ± 0,9	3,4 ± 1,0
p ^††^		<0,001	0,697	0,018
**Idade (anos) - *r* (p)** ^‡‡^		-0,301 (p<0,001)	-0,049 (0,529)	-0,133 (p=008)
**Escolaridade**				
Não estudou/Ensino Fundamental	92	2,2 ± 0,7	3,8 ± 0,9	3,4 ± 1,0
Ensino Médio	40	2,7 b ^¶^ ± 0,6	3,9 ± 0,8	3,7 ± 0,9
Ensino Superior	34	3,0 a ± 0,6	4,0 ± 0,9	3,8 ± 0,8
p ^††^		<0,001	0,667	0,094
**Sabe ler**				
Sim	158	2,5** ± 0,7	3,9 ± 0,9	3,5 ± 1,0
Não	8	1,9 ± 0,8	4,1 ± 0,7	3,7 ± 1,2
P ^§§^		0,010	0,532	0,594
**Sabe escrever**				
Sim	159	2,5** ± 0,7	3,8 ± 0,9	3,5 ± 1,0
Não	7	1,9 ± 0,9	4,2 ± 0,6	4,1 ± 0,7
p ^§§^		0,033	0,296	0,129

*CA = Avaliação das Informações de Saúde; ^†^AE = Capacidade de Interagir Ativamente com os Profissionais de Saúde; ^‡^NHS = Navegar no Sistema de Saúde; ^§^HLQ = *Health Literacy Questionnaire*; ^||^Média ± DP = Desvio-padrão; ^¶^Diferença estatística significativa a 1%, estimada pelo teste de Análise de Variância (*One Way*) – *post hoc* Bonferroni; **Diferença estatística significativa a 5%; ^††^Análise de Variância (*One Way*) – *post hoc* Bonferroni; “a” Escore médio significativamente mais elevado, em relação às demais médias da escala específica; “b” escore médio intermediário, que difere significativamente da média mais elevada, bem como da menor média; “c” média significativamente menor, em comparação às demais médias da escala específica; ^‡‡^r(p)Correlação de Pearson; ^§§^Teste t-Student para grupos independentes

Ao se comparar as faixas etárias e as escalas Avaliação das Informações de Saúde (CA), Capacidade de Interagir Ativamente com os Profissionais de Saúde (AE) e Navegar no Sistema de Saúde (NHS), observou-se diferença estatística significativa para a escala Avaliação das Informações de Saúde (CA) (p<0,001). Identificou-se escore médio mais elevado nas faixas etárias até 49 anos e de 50 a 69 anos. Ressalta-se que a definição da escala CA com escore alto indica que a pessoa é capaz de identificar boas informações e fontes confiáveis, resolver as informações conflitantes sozinha ou com ajuda de outras pessoas. Esta capacidade se mostrou presente nos pacientes com até 49 anos e de 50 a 69 anos (Tabela 4).

Ao comparar a idade dos pacientes às escalas CA, AE, NHS obteve-se correlação estatisticamente significativa e negativa para a escala CA (r = -0,301; p<0,001). Isso aponta que os elevados escores na escala Avaliação das Informações de Saúde (NHS) devem estar correlacionados aos pacientes com menores faixas etárias da amostra (Tabela 4).

A escala Navegar no Sistema de Saúde (NHS) (p=0,018) mostrou resultado significativamente superior, com escore médio na faixa etária de 50 a 69 anos, quando comparada aos demais. Na escala de Capacidade de Interagir Ativamente com os Profissionais de Saúde (AE), não foi observada diferença estatística significativa. Também os resultados das correlações entre a idade e as escalas AE e NHS não mostraram diferenças estatísticas significativas. Ao considerar a comparação da escolaridade em relação às escalas CA, AE, e NHS, encontrou-se diferença estatística significativa com a escala CA (p<0,0001). Pacientes com níveis de escolaridade de Ensino Médio e Ensino Superior Completo apresentaram escores médios significativamente mais elevados, quando comparados àqueles com menor nível. Nas escalas AE e NHS não se encontraram diferenças estatísticas significativas (Tabela 4).

Em relação ao fato de saber ler, obteve-se diferença significativa, com destaque exclusivamente na escala CA (2,5±0,7 vs 1,9± 0,9 p=0,010), na qual os pacientes que referiram saber ler apresentaram escore médio significativamente superior, em comparação aos demais.

Um resultado semelhante foi observado em relação à variável saber escrever, quando comparada às escalas CA, AE, NHS. A diferença significativa foi obtida pela escala CA (p=0,032), apontando que os pacientes que declararam saber escrever apresentaram escore médio significativamente superior (Tabela 4).

Ao considerar as análises que envolveram as comparações da faixa etária, na escala Capacidade de Encontrar Boas Informações de Saúde (FHI), houve uma diferença estatística significativa (p=0,002), e apontou-se que os escores médios nas faixas de idade até 49 anos e de 50 a 59 anos se mostraram significativamente mais elevados. Também se encontrou resultado semelhante na comparação da faixa etária com a escala Compreender as Informações de Saúde bem o Suficiente para Saber o que Fazer (UHI) (p=0,001) (Tabela 5).

**Tabela 5 -:** Média e desvio-padrão para as escalas FHI* e UHI^†^ da escala HLQ^‡^, segundo as características sociodemográficas. Ijuí, RS, Brasil, 2023

**Variáveis**	n	**HLQ** ^‡^	
		FHI*	**UHI** ^†^
		**Média ± DP** ^§^	**Média ± DP** ^§^
**Faixa etária (anos)**			
Até 49	17	3,5 a ^¶^ ± 1,1	3,5 a ^¶^ **± 1,1**
50 a 69	52	3,4 ^¶^ ± 0,8	**3,5** ^¶^ **± 0,7**
Superior a 69	97	2,9 ± 1,0	3,0 **± 0,9**
p ^||^		0,002	0,001
Idade (anos) - *r* (p)**		-0,334 (p<0,001)	-0,301 (p<0,001)
**Escolaridade**			
Não estudou/Ensino Fundamental	92	2,8 ± 0,9	3,0 **± 0,9**
Ensino Médio	40	3,5 a ^¶^ **± 0,9**	3,3 b ^¶^ **± 0,8**
Ensino Superior	34	3,8 b **± 0,8**	3,6 a ^¶^ **± 1,0**
p ^||^		**<** 0,001	0,002
**Sabe ler**			
Sim	158	3,2 ^††^ **± 1,0**	3,2 ^††^ **± 0,9**
Não	8	2,4 **± 0,7**	2,0 **± 0,5**
p ^‡‡^		0,008	**<** 0,001
**Sabe escrever**			
Sim	159	3,2	3,2 ^††^
Não	7	2,5	2,0
p ^‡‡^		0,071	0,001

*FHI = Capacidade de Encontrar Boas Informações de Saúde; **†**UHI = Compreender as Informações de Saúde Bem o Suficiente para Saber o que Fazer; ^‡^HLQ = *Health Literacy Questionnaire*; ^§^Média±DP = Desvio-padrão; ^||^Análise de Variância (*One Way*) – *post hoc*; Sobre os resultados com diferença estatisticamente significativa tem-se a notação: “a” escore médio significativamente mais elevado, em relação às demais médias da escala específica; “b” escore médio intermediário, que difere significativamente da média mais elevada, bem como da menor média; “c” média significativamente menor, em comparação às demais médias da escala específica; ^¶^Diferença estatística significativa a 1%, estimada pelo teste de Análise de Variância (*One Way*) – *post hoc* Bonferroni; **r(p)= Correlação de Pearson; ^††^Diferença estatística significativa a 5%; ^‡‡^Teste t-Student para grupos independentes

Segundo definição da escala, há evidências de que as faixas etárias até 49 anos e de 50 a 69 anos apresentaram maior habilidade de explorar as informações. Além disso, as duas faixas etárias também apresentaram maior capacidade de entender as informações escritas em relação à saúde.

Ao correlacionar as escalas com a idade, identificaram-se as correlações estatisticamente significativas e negativas, tanto entre a idade e a escala FHI (r = -0,334; p<0,001), quanto sobre os dados da escala UHI (r = -0,301; p<0,001). Há evidências de que os pacientes com as idades mais elevadas se mostraram correlacionados aos menores escores.

No que se refere às comparações sobre a questão saber ler, nos escores das escalas FHI e UHI obteve-se algumas diferenças estatisticamente significativas. Identificou-se que o escore médio foi significativamente maior entre os investigados que declararam saber ler (FHI) (p=0,010). Um resultado semelhante ocorreu ao comparar a variável sabe ler. Ao se considerar as comparações com a escala UHI, sobre a questão saber escrever (p=0,010), a diferença estatística significativa se configurou exclusivamente sobre esta escala, apontando um escore médio superior no grupo que relatou saber escrever.

## Discussão

Este é o primeiro estudo brasileiro que avaliou o LS de pacientes com DCNTs e sua relação com as variáveis sociodemográficas (idade, escolaridade, saber ler e escrever, e se faz parte da Medicina Preventiva) e clínicas (tempo de diagnóstico de DCNTs) por meio da análise bivariada. Identificou-se que os maiores escores foram nas escalas: Suporte Social Para a Saúde e Capacidade de Interagir Ativamente com os Profissionais de Saúde, ambos os resultados semelhantes a outro estudo internacional, com pessoas de uma comunidade de pesca rural no norte do Egito ^( 13 )^.

Predominaram as pessoas com Doenças Cardiovasculares (DCV,) semelhante a um estudo que avaliou o LS no Brasil com pessoas acometidas por DCNTs, ao qual predominou as DCV com 230 pessoas (29%) e o sexo feminino (67,5%) ^( 10 )^. Outros estudos recentes também reafirmaram esses resultados ^( 1 , 6 )^. Observou-se uma redução do LS com o aumento da idade e menor escolaridade. O LS desempenha papel significativo no manejo das DCNTs, consideradas complexas, que se tornam um desafio significativo e de longo prazo para as pessoas e para o sistema de saúde ^( 4 , 6 )^.

Os menores escores foram encontrados nas escalas Avaliação das Informações de Saúde e Capacidade de Encontrar Boas Informações de Saúde, o que corrobora outros estudos, realizados no Brasil, que utilizaram o mesmo instrumento com pessoas com HIV e com doença renal crônica ^( 12 , 14 )^.

Neste estudo, os pacientes com idades avançadas e escolaridade baixa tiveram os melhores desempenhos na escala Suporte Social Para a Saúde, resultado semelhante ao encontrado em outros estudos ^( 15 )^. Ademais, o suporte social de qualidade promove melhores resultados de saúde ^( 16 )^ e da autogestão ^( 17 )^. Para tanto, a educação e apoio aos familiares e cuidadores envolvidos nesse contexto pode ser uma estratégia útil para os serviços de saúde; outros estudos ^( 18 - 19 )^ sugerem que os cuidadores de pessoas com DCNTs têm necessidade de informações sobre os cuidados, sendo cruciais os princípios do LS no cotidiano dos cuidadores, profissionais e gestores para os melhores desfechos e decisões em saúde ^( 11 )^.

Um estudo realizado na Austrália apontou que os pacientes com idade avançada e grau de instrução menor têm necessidade e/ou recebem maior Suporte Social Para a Saúde, o que favorece o LS desta população ^( 20 )^. Outra pesquisa no Brasil mostrou resultados semelhantes aos dessa investigação ^( 14 , 21 )^.

É fundamental que os profissionais de saúde avaliem o nível de LS dos pacientes e adaptem as informações e o apoio às competências de LS conforme o contexto dos seus pacientes, de forma singular, pois favorece a gestão da sua condição crônica de saúde durante a vida ^( 9 )^. Ressalta-se que as estratégias pautadas em comunicação assertiva, informações compreensíveis e o acesso aos serviços de forma clara favorecem o LS das pessoas ^( 7 , 22 )^.

Dados deste estudo evidenciaram que os pacientes que participavam de um programa de Medicina Preventiva tiveram melhor média na escala Informações Suficientes Para Cuidar da Saúde. Ou seja, sentiam-se confiantes de que tinham as informações de que precisavam para conviver, gerenciar suas condições e tomar as decisões em relação à sua saúde ^( 8 )^, diferente de outros estudos que apresentaram médias mais baixas ^( 12 - 13 )^. Isso ressalta a importância de programas que sejam baseados nos princípios de LS ^( 3 )^.

É importante ter relação estabelecida com pelo menos um profissional de saúde que os conheça bem e em quem confiem, para receber informações úteis para os ajudar a compreender as orientações e a tomar decisões sobre sua saúde ^( 8 )^. O fortalecimento do vínculo facilita a continuidade do cuidado prestado e oportuniza o desenvolvimento de estratégias de forma singular. Considera-se estratégico que a transmissão de informações pelos profissionais de saúde seja clara e com linguagem acessível, de maneira a favorecer a compreensão e incentivar o autogerenciamento ^( 23 )^, visto que os serviços de saúde constituem a porta de entrada principal, e são considerados como fonte de informação segura para os usuários.

Os pacientes do estudo em tela apontaram fragilidades nas escalas Informações Suficientes Para Cuidar da Saúde e Avaliação das Informações de Saúde. Apresentaram lacunas em relação ao seu conhecimento de saúde, não conseguiam compreender as informações que recebiam e acreditavam não possuir informações necessárias para o autogerenciamento. Essa fragilidade foi corroborada por outro estudo ^( 12 )^, que identificou o grau de adesão baixo/insuficiente ao tratamento medicamentoso de pessoas com HIV, devido às fragilidades encontradas sobre Informações Suficientes Para Cuidar da Saúde e Avaliação das Informações de Saúde.

Esses resultados têm implicações importantes e reafirmam a necessidade, e avanços, no cuidado às pessoas com DCNTs, em termos de comunicação assertiva entre os pacientes e os profissionais de forma singular, em concordância com outros estudos que apresentaram resultados semelhantes ^( 11 , 24 - 25 )^. O acesso às informações não é o principal problema, mas sim a sua qualidade, visto que as pessoas precisam entender as orientações de forma clara para seu autogerenciamento. Para tanto, a disponibilização de materiais educativos sobre a doença e o tratamento em linguagem acessível e de fonte segura são recursos que podem ser adotados ^( 7 , 23 )^.

A comunicação assertiva pode ser empregada por meio do uso de *teach back*, que favorece a compreensão das informações ^( 26 )^. Essas estratégias oferecem suporte para que as pessoas aumentem o conhecimento, a capacidade para a tomada de decisão compartilhada, o engajamento e o seu autogerenciamento ^( 26 - 27 )^.

Este estudo identificou que os pacientes mais jovens e com maior grau de instrução tiveram potencialidades de LS para identificar as boas informações em fontes seguras, bem como entender as informações conflitantes sozinhas ou com a ajuda de outras pessoas. O nível de escolaridade é relevante, uma vez que as pessoas com grau de escolaridade maior podem obter melhor acesso às informações sobre os cuidados em saúde e o tratamento das DCNTs ^( 28 )^.

Isso, embora influencie, nem sempre é considerado característica determinante, visto que pessoas com níveis altos de estudo podem ter LS insuficiente, pois este é singular e transcende o saber ler ou escrever, consideradas as habilidades de tomada de decisão, promoção da saúde, acesso e navegação no sistema de saúde ^( 7 )^.

Os resultados da escores da escala Cuidado Ativo da Saúde mostraram fragilidades para o LS. Ela relacionou-se com a responsabilidade do indivíduo com sua saúde e com envolver-se proativamente de modo a tomar suas próprias decisões. Este fato pode estar associado à idade e à baixa escolaridade, pois a idade avançada pode reduzir a capacidade de realizar tarefas cognitivas que requeiram o processamento de informações. As pessoas com maior idade parecem ter mais dificuldade para concluir tarefas que exijam raciocínio ou tirar conclusões a partir das informações apresentadas ^( 29 )^.

Nesse sentido, o suporte social deve ser uma preocupação dos profissionais de saúde, em especial da Enfermagem, a fim de aprimorar as práticas educativas que possam proporcionar uma escuta qualificada, com esclarecimento sobre os cuidados, e considerando, inclusive, as questões emocionais ^( 30 )^, com vistas à abordagem integral aos idosos e com maior vulnerabilidade.

Ao se considerar os escores das escalas Capacidade de Encontrar Boas Informações de Saúde e Compreender as Informações de Saúde bem o Suficiente para Saber o que Fazer, identificou-se que, até a faixa etária de 69 anos, as pessoas apresentaram potencialidades de LS. É necessário que as pessoas consigam explorar, compreender e utilizar diferentes fontes de informações, sendo capazes de escrever adequadamente em formulários, quando necessário.

O estudo possibilitou a identificação dos aspectos que necessitam ser aprimorados na assistência, a fim de subsidiar a elaboração de estratégias e favorecer o LS. Com o estudo, foi possível perceber que o investimento em políticas de saúde, nos âmbitos público e privado, baseadas em estratégias de LS é crucial, assim como se faz necessário apoiar os gestores, profissionais da saúde e os usuários engajados para que haja a mudança efetiva.

Este estudo tem algumas limitações: o tipo de estudo eleito, quantitativo e transversal, que não permite estabelecer relação de causalidade entre as variáveis, bem como a sua realização em um único hospital geral. Portanto, deve-se ter cuidado ao generalizar os resultados. Apesar das limitações, este estudo traz inovações sobre a importância de considerar o LS para a qualificação da assistência em saúde em DCNTs. Os resultados podem fornecer subsídios para o aprofundamento de pesquisas futuras investigando a relação causal entre o LS e as variáveis sociodemográficas e clinicas dos pacientes internados com DCNTs.

## Conclusão

Os pacientes apresentaram potencialidades em LS, nas escalas Suporte Social Para a Saúde (3,5±0,4), Capacidade de Interagir Ativamente com os Profissionais de Saúde (3,9±0,9) e Navegar no Sistema de Saúde (3,6±1,0). Em contrapartida, identificaram-se fragilidades na Avaliação das Informações de Saúde. (2,5±1,0) e na Capacidade de Encontrar Boas Informações de Saúde (3,1±1,0).

Houve uma associação significativa entre o LS e as variáveis sociodemográficas, como idade, escolaridade e saber ler e escrever. Os pacientes na faixa etária até 49 anos e de 50 a 59 anos apresentaram médias menores, enquanto aqueles com menor escolarização apresentaram médias maiores na escala Suporte Social Para a Saúde. As faixas etárias menores evidenciaram os melhores resultados de LS nas escalas: Avaliação das Informações em Saúde, Capacidade de Encontrar Boas Informações de Saúde e Compreender as Informações de Saúde Bem o Suficiente para Saber o que Fazer.

Os pacientes mais jovens, com maior escolaridade e habilidades de leitura e escrita, apresentaram escores mais elevados em escalas relacionadas à avaliação e compreensão das informações de saúde. Por outro lado, os indivíduos de idade mais avançada e menor escolaridade mostraram escores menores, indicando as fragilidades na compreensão das informações em saúde. Por outro lado, a escala Suporte Social Para a Saúde se mostrou como potencialidade e pode favorecer o LS na avaliação e na utilização dos serviços de saúde.

Os pacientes que participavam do programa de Medicina Preventiva tiveram maiores médias na escala Informações Suficientes Para Cuidar da Saúde. As pessoas com mais tempo de DCNTs informaram que são mais compreendidas e apoiadas pelos profissionais de saúde.

Diante do exposto, é necessário que os profissionais de saúde reflitam e implementem as estratégias de LS de forma singular para os pacientes com faixa etária maior e baixa escolaridade, a fim de que estas possam usufruir na tomada de decisão sobre a sua saúde e o seu bem-estar.

# Translation: Características sociodemográficas y clínicas asociadas a la alfabetización en salud en personas hospitalizadas por enfermedades crónicas*

**DOI:** 10.1590/1518-8345.7395.4493

**Authors:** Francini de Oliveira Rodrigues, Eliana Elisa Rehfeld Gheno, Letícia Y Castro, Arnaldo Nogaro, Amanda Caroline Mélo da Rosa, Adriane Cristina Bernat Kolankiewicz

**Affiliations:** 1 Universidade Regional do Noroeste do Estado do Rio Grande do Sul, Ijuí, RS, Brasil; 2 Universidade do Alto Uruguai e das Missões, Campus Erechim, RS, Brasil; 3 Becaria del Conselho Nacional de Desenvolvimento Científico e Tecnológico (CNPq), Brasil

**Keywords:** Alfabetización en Salud, Enfermedades no Transmisibles, Hospitalización, Enfermedades Crónicas, Acceso a la Información, Gestión en Salud

## Abstract

identificar la asociación entre alfabetización en salud y las variables sociodemográficas y clínicas de pacientes hospitalizados con enfermedades crónicas.

estudio transversal, realizado con pacientes con enfermedades crónicas ingresadas en un hospital general. Se utilizó un cuestionario sociodemográfico/clínico y el *Health Literacy Questionnaire*, compuesto por nueve escalas, medidas por puntajes. Análisis descriptivo e inferencial de los datos.

el apoyo social a la salud se asoció con el nivel de educación primaria (p=0,009). La comprensión y el apoyo de los profesionales de la salud se relacionaron con la duración de la enfermedad crónica (p=0,044). La evaluación de la información de salud se asoció con la franja etaria (p=0,001), saber leer (p=0,010) y escribir (p=0,032). Navegar por el sistema de salud también estuvo relacionado con la franja etaria (p=0,018), al igual que la capacidad para encontrar buena información de salud (p=0,002), saber leer (p=0,010) y comprender la información de salud lo suficientemente bien como para saber qué hacer se asoció con la franja etaria (p=0,001) y saber escribir (p=0,010).

la educación, la franja etaria, la duración de la enfermedad crónica y el saber leer y escribir interfieren en la alfabetización en salud. Esto destaca que es necesario desarrollar estrategias personalizadas que consideren esas variables para mejorar la alfabetización en salud de la población hospitalizada.

## Destacados

**(1)** Primer estudio brasileño que evaluó la alfabetización en salud de personas con ECNT. **(2)** Potencialidades: Capacidad para interactuar, navegar en el sistema y apoyo social. **(3)** Debilidades: Evaluación de la información de salud y capacidad para encontrarla.**(4)** La edad, la educación y el saber leer y escribir interfieren en la alfabetización en salud.

## Introducción

Las enfermedades crónicas no transmisibles (ECNT) son un problema de salud pública mundial. Constituyen la principal causa de discapacidad y mortalidad prematura en el mundo y son responsables de la muerte de 40,8 millones de personas, lo que equivale al 74% de todas las muertes ^( 1 )^.

Padecer ECNT implica tener acceso a bienes y servicios públicos, derechos garantizados, información, empleo, ingresos y la posibilidad de tomar decisiones beneficiosas para la salud ^( 2 )^. En este contexto, la Alfabetización en Salud (AS) contribuye y puede modificar factores de salud negativos y ayudar a comprender las fortalezas y debilidades de las personas y las comunidades. Representa el conocimiento y las competencias personales que se acumulan a través de las actividades cotidianas, las interacciones sociales y las generaciones. Está mediada por estructuras organizativas y por la disponibilidad de recursos que les permitan a las personas acceder, comprender, evaluar y utilizar información y servicios, de manera que promuevan y mantengan la buena salud y el bienestar ^( 3 )^.

La AS se considera el mejor indicador de salud en relación a muchos determinantes sociales ^( 4 )^. Una AS deficiente provoca el uso inadecuado de los servicios y genera resultados de salud negativos. Además, se asocia con altas tasas de hospitalización, mayor prevalencia de enfermedades, menor uso de métodos preventivos y baja adherencia a los tratamientos. Por otro lado, cuando es adecuada, mejora la autogestión de la atención, la calidad de vida (CV) y, por ende, reduce los reingresos hospitalarios, la sobrecarga y los costos ^( 5 )^.

En este caso, los pacientes cuya salud empeora debido a las ECNT a menudo requieren hospitalización, debido a su complejidad clínica. Y los profesionales de la salud que les brindan atención, especialmente los enfermeros, deben contribuir para preparar el alta de forma segura y de modo que se le dé continuidad a la atención. Para ello, es necesario que el paciente desarrolle habilidades, dependiendo de su nivel de AS, para facilitar la gestión, el empoderamiento, que comprenda su autocuidado y, por consiguiente, evitar eventos adversos y reingresos ^( 6 )^.

Los estudios se han limitado a evaluar habilidades cognitivas de lectura y aritmética, y no consideran otros aspectos de la AS que les permiten a los individuos y a la sociedad ser responsables de sus condiciones de salud ^( 7 )^. Investigaciones con personas afectadas por ECNT en Etiopía identificaron que los niveles de AS varían según las características sociodemográficas y clínicas. Por ello, los profesionales de la salud deben evaluar el nivel de AS y adaptar la información y el apoyo a las habilidades de AS según el contexto del paciente, lo que favorece el manejo de su condición crónica a lo largo de su vida ^( 8 )^.

En el contexto internacional, hay estudios que identificaron la relación entre la AS y la caracterización sociodemográfica ^( 9 )^, pero no las condiciones clínicas. En Brasil, se realizaron estudios para evaluar multidimensionalmente la AS en diferentes poblaciones, como profesionales de la salud, usuarios generales, cuidadores domiciliarios y personas portadoras del virus de inmunodeficiencia humana (VIH) ^( 10 - 12 )^. En Brasil, la AS es poco estudiada en el contexto de las prácticas y la gestión en salud, no hay una estandarización de estrategias y planes efectivos para lograr una mejor AS y efectividad, y que puedan ser aplicados durante el período de internación, dado que son pocas las instituciones que realizan acciones enfocadas en los mismos, para contribuir a reducir los reingresos hospitalarios.

Por lo tanto, al identificar las asociaciones entre la AS y las variables sociodemográficas y clínicas, los profesionales de enfermería y las instituciones de salud podrán reconocer las debilidades de la AS y desarrollar estrategias efectivas para las personas con enfermedades crónicas, con el fin de reducir los reingresos. Se sabe que hay pocos estudios nacionales e internacionales que relacionan la AS con la caracterización sociodemográfica y clínica desde la perspectiva de las personas que viven con ECNT. Debido a este contexto y a la laguna de conocimiento identificada, se justifica la realización del presente estudio.

El objetivo general del estudio fue identificar la asociación entre la alfabetización en salud y las variables sociodemográficas y clínicas de pacientes hospitalizados con enfermedades crónicas.

## Método

### Tipo de estudio

Estudio cuantitativo y transversal basado en las directrices del *Strengthening the Reporting of Observational Studies in Epidemiology* (STROBE). Cabe señalar que la AS en el contexto hospitalario será analizada desde la perspectiva de pacientes con enfermedades crónicas, en función de las variables sociodemográficas y clínicas.

### Contexto y recolección de datos

El estudio fue realizado en un hospital general que cuenta con 76 camas en unidades de internación clínica, en la ciudad de Ijuí, RS, Brasil. Las unidades se componen de 18 camas privadas y 58 camas no privadas dobles. La institución cuenta con un programa de “Medicina Preventiva en la Atención Integral de Salud”, que hace un seguimiento del paciente mediante actividades de atención integral y preparación para el alta hospitalaria.

### Periodo

Recopilación de datos realizada entre abril y octubre de 2023.

### Participantes

Participaron 166 pacientes hospitalizados con ECNT.

### Criterios de selección

Criterios de inclusión: personas mayores de 18 años, con diagnóstico de ECNT registrado en la historia clínica, hospitalizadas por al menos 24 horas. Se excluyó a las parturientas, los pacientes quirúrgicos, pacientes institucionalizados luego del alta y las personas con actividad cognitiva deteriorada, que fue evaluada por la primera autora, enfermera, a través de los registros en la historia clínica y presencialmente a pie de cama, a partir de la evaluación del nivel de conciencia del paciente y del contenido.

### Definición de la muestra

La población de estudio estuvo compuesta por pacientes hospitalizados con ECNT en la institución, de abril a agosto de 2023, que alcanzó un tamaño poblacional de aproximadamente 370 pacientes, lo que caracteriza una población finita. Para estimar la base de cálculo del tamaño de la muestra, se consideró como factor principal del estudio la escala HLQ. Se asumió como total de la escala un puntaje medio de 3,0 (±1,0), que define el punto medio.

Basándose en esa estimación y asumiendo un nivel de significación del 5% (α=0,05), un margen de error del 7,5% sobre la media a estimar, lo que equivale a una variación de 0,2 unidades, se calculó que el tamaño mínimo de la muestra era de 159 pacientes. Aplicando el factor de corrección para poblaciones finitas, se estimó que el tamaño mínimo necesario para este estudio era de 112 participantes. De esta manera, al llegar a 166 participantes, se cumplió con un tamaño de muestra que alcanzó representatividad muestral.

Las historias clínicas electrónicas fueron analizadas diariamente para identificar el perfil clínico del paciente y el tiempo de estadía, y se los invitó a participar durante el período de recolección de datos. Se invitó a 192 pacientes y, de ellos, 26 rechazaron la invitación. Así, la muestra estuvo compuesta por 166 pacientes hospitalizados con ECNT.

### Variables de estudio

Se consideraron las variables sociodemográficas edad, sexo, ingreso, escolaridad, raza, estado civil, ser parte del programa de medicina preventiva, saber leer y escribir. Y variables clínicas como: condiciones de salud, tiempo de diagnóstico, tener una o más enfermedades crónicas. Para el LS se utilizaron las escalas del cuestionario *Health Literacy Questionnaire* (HLQ).

## Instrumentos utilizados para recolectar información

La información sobre las condiciones clínicas de los pacientes se obtuvo en el expediente sanitario. Para las variables sociodemográficas y HL se utilizó un cuestionario sociodemográfico y el HLQ, respectivamente, a pie de cama. El HLQ es un instrumento multidimensional para medir HL ^( 7 )^, validado para su uso en Brasil ^( 10 )^.

El HLQ consta de 44 ítems y está subdividido en dos partes. La parte 1, está compuesta por los siguientes dominios: 1. Comprensión y apoyo de los profesionales de la salud (HPS); 2. Información suficiente para cuidar su salud (HSI); 3. Cuidado activo de la salud (AMH); 4. Apoyo social a la salud (SS); 5. Evaluación de la información de salud (CA). En estas escalas, el puntaje para calcular la media oscila entre 1 y 4, y las respuestas van desde “totalmente en desacuerdo” hasta “totalmente de acuerdo” ^( 7 , 10 )^.

En la parte 2 están las escalas: 6. Capacidad para interactuar activamente con los profesionales de la salud (AE); 7. Navegar por el Sistema de Salud (NHS); 8. Capacidad para encontrar buena información de salud (FHI); 9. Comprender la información de salud y saber qué hacer (UHI). Estas escalas se evalúan con un puntaje que puede variar del 1 al 5, y las respuestas van desde “siempre difícil” hasta “siempre fácil” ^( 7 , 10 )^.

El HLQ no proporciona una puntuación global, sino puntajes para cada una de las escalas por separado. La puntuación indica las fortalezas y necesidades de cada persona con respecto a la AS ^( 7 , 10 )^. El cálculo se realiza sumando cada ítem de la escala y este valor se divide por el número de ítems de la escala, y el valor se presenta como la media de la puntuación. El estudio obtuvo el consentimiento previo de los autores para utilizar el instrumento debido a que tiene derechos de autor (hl-info@swin.edu.au)

## Recolección de datos

Los instrumentos se aplicaron a pie de cama, cara a cara, mediante un teléfono celular o *tablet*. Cada dos días se accedía a la historia clínica electrónica del paciente para comprobar si había nuevos ingresos. Posteriormente, el entrevistador se acercaba a la cama de la persona y la invitaba a participar en el momento y, si no era posible, el asistente salía de la habitación y regresaba más tarde. Las personas respondieron el cuestionario en ausencia de un cuidador o familiar, para garantizar la privacidad y cumplir con los aspectos éticos. En la recolección de datos participaron cuatro asistentes de investigación, estudiantes de enfermería y medicina, previamente capacitados por la coordinadora del proyecto, última autora, mediante lectura y seguimiento en la recolección inicial de datos, y seguimiento hasta que finalizó. Las entrevistas duraron aproximadamente 30 minutos.

## Tratamiento y análisis de los datos

Los resultados se presentaron mediante estadística descriptiva, utilizando medidas de tendencia central y variabilidad. La simetría de las distribuciones continuas se estudió mediante la prueba de Kolmogorov-Smirnov. Para verificar la confiabilidad del instrumento se calculó el alfa de Cronbach. Para comparar las variables continuas entre dos grupos independientes se utilizó la prueba t de Student. Cuando la comparación de los puntajes involucró a tres o más grupos independientes, se utilizó el Análisis de Varianza (Unidireccional) – *post hoc* Bonferroni. La relación de linealidad entre las edades y los puntajes de las escales se analizó mediante el coeficiente de correlación de Pearson. El tratamiento estadístico se realizó con ayuda del *Statistical Package for the Social Sciences* versión 25.0 (SPSS Inc., Chicago, IL, USA, 2018) para Windows y se adoptó un nivel de significación del 5% para los criterios de decisión estadística.

## Aspectos éticos

El estudio respetó los aspectos éticos de acuerdo con la Resolución n° 466/2012. La investigación fue aprobada el 28 de febrero de 2023 por el Comité de Ética en Investigación, con número 66693823.7.0000.5350 y Dictamen nº 5.915.197.

## Resultados

De los 166 pacientes con ECNT, las enfermedades más prevalentes fueron cardiovasculares, 40 (24,1%), metabólicas, 41 (24,7%), neurológicas y 38 (22,9%), neoplasias. Hubo predominio de pacientes del sexo femenino (87; 52,4%), con media de edad de 69,3 ±14,3 años, blancos (159; 95,8%), con ingresos de hasta cinco salarios mínimos (121; 73%). La mayoría (158; 95,8%) refirió saber leer y escribir. En cuanto a la educación, (92;55,4%) pacientes no estudiaron o tenían educación primaria, (40; 24,1%) educación secundaria y (34; 20,5%) educación superior. Respecto a su participación en el programa de Medicina Preventiva, la mayoría refirió no participar (117; 70,5%). Respecto al tiempo con la enfermedad crónica, la mayoría respondió que tenía la enfermedad desde hacía más de 14 años (70; 42,2%) (Tabla 1).

**Tabla 1 -:** Caracterización sociodemográfica y clínica de personas que viven con Enfermedades Crónicas No Transmisibles. Ijuí, RS, Brasil, 2023

**Variables**	**Total la muestra** **(n=166)**	
	**n**	**%**
**Sexo**		
Femenino	87	52,4
Masculino	79	47,6
**Franja etaria (años)**		
Hasta 49	17	10,2
50 a 69	52	31,3
Más de 69	97	58,4
**Edad (años)**		
Media ± DE*	69,3±14,3	
**Color**		
Blanco	159	95,8
No blanco	7	4,2
**Estado civil**		
Vive en unión estable	107	64,5
No vive en unión	59	35,5
**Ingreso familiar mensual**		
Hasta 2 SM ^†^	30	18,1
3 a 5 SM ^†^	65	39,2
6 a 10 SM ^†^	30	18,1
Más de 10 SM ^†^	15	9,0
No responde	26	15,7
**Sabe leer**		
No	8	4,2
Sí	158	95,2
**Sabe escribir**		
No	7	4,3
Sí	159	95,7
**Educación**		
No estudió/ Educación primaria	92	55,4
Educación secundaria	40	24,1
Educación superior completa	34	20,5
**Programa de Medicina Preventiva**		
No	117	70,5
Sí	49	29,5
**Tiempo con ECNT** ^‡^		
Hasta 1 año	19	11,4
1 a 5 años	34	20,5
5 a 14 años	43	25,9
Más de 14 años	70	42,2

*Media ± DE = Desviación estándar; ^†^SM = Salario mínimo R$1302,00, Brasil, 2023; ^‡^ENCT = Enfermedades crónicas no transmisibles

En la Tabla 2 se presentan las medias del HLQ y la consistencia interna del instrumento

**Tabla 2 -:** Medidas de tendencia central, variabilidad y consistencia interna de las escalas del HLQ*. Ijuí, RS, Brasil, 2023

Escalas HLQ*	**Media**	**Desviación estándar**	αC ^†^
**Parte 1- Puntaje de 1-4**			
1. Comprensión y apoyo de los profesionales de la salud	3,3	0,5	0,778
2. Información suficiente para cuidar su salud	2,8	0,6	0,821
3. Cuidado activo de la salud	2,9	0,5	0,658
4. Apoyo social a la salud	3,5	0,4	0,741
5. Evaluación de la información de salud	2,5	0,7	0,813
**Parte 2- Puntaje de 1-5**			
6. Capacidad para interactuar activamente con los profesionales de la salud	3,9	0,9	0,860
7. Navegar en el sistema de salud	3,6	1,0	0,870
8. Capacidad para encontrar buena información de salud	3,1	1,0	0,825
9. Comprender la información de salud y saber qué hacer	3,2	0,9	0,787

*HLQ = *Health Literacy Questionnaire*; ^†^αC = Coeficiente alfa de Cronbach

Al evaluar los puntajes del LS, se identificó que las escalas que presentaron mejores promedios, consideradas fortalezas, fueron: Capacidad para interactuar activamente con los profesionales de la salud, Navegación en el sistema de salud y Apoyo social para la salud, por lo tanto, consideradas fortalezas. Las escalas Evaluación de la información de salud, Información suficiente para cuidar la salud y Capacidad para encontrar buena información de salud, con promedios más bajos, consideradas debilidades (Tabla 2).

Al comparar los grupos de edad con las escalas Comprensión y Apoyo de los Profesionales de la Salud (HPS); Información Suficiente para el Cuidado de la Salud (HSI); Atención Activa de la Salud (AMH); Apoyo Social para la Salud (SS), se encontró que no hubo diferencias estadísticamente significativas. Se encontró una correlación débil pero significativa en la escala de Apoyo Social para la Salud (SS) con la mayor edad de los participantes del estudio (r=0,153; p=0,049) (Tabla 3).

**Tabla 3 -:** Media y desviación estándar de las escalas HPS*, HSI^†^, AMH^‡^ y SS^§^ de la escala HLQ^||^, según las características específicas del perfil de la muestra. Ijuí, RS, Brasil, 2023

**Variables**	**N**	**HLQ** ^||^			
		HPS*	**HSI** ^†^	**AMH** ^‡^	**SS** ^§^
		**Media ± DE** ^¶^	**Media ± DE** ^¶^	**Media ± DE** ^¶^	**Media ± DE** ^¶^
**Franja etaria (años)**					
Hasta 49	17	3,3 ± 0,4	2,7 ± 0,6	2,9 ± 0,4	3,5 ± 0,5
De 50 a 69	52	3,3 ± 0,5	2,8 ± 0,6	3,0 ± 0,5	3,5 ± 0,5
Más de 69	97	3,4 ± 0,5	2,8 ± 0,7	2,9 ± 0,5	3,6 ±0,4
P **		0,793	0,689	0,227	0,381
**Edad (años) -** r **(p)** ^††^		0,094 (p=0,226)	0,087 (p=0,265)	-0,048 (p=0,539)	0,153 (p=0,049)
**Educación**					
No estudió/Educación primaria	92	3,3 ± 0,5	2,8 ± 0,7	2,9 ± 0,5	3,6a ^‡‡^ ± 0,4
Educación secundaria	40	3,4 ± 0,5	2,8 ± 0,6	3,0 ± 0,4	3,5ab ± 0,4
Educación Superior	34	3,4 ± 0,5	2,7 ± 0,6	2,9 ± 0,5	3,4b ± 0,5
p **		0,898	0,793	0,892	0,009
**Sabe leer**					
Sí	158	3,3 ± 0,5	2,8 ± 0,6	2,9 ± 0,5	3,5 ± 0,4
No	8	3,4 ± 0,7	2,8 ± 1,1	2,9 ± 0,6	3,7 ± 0,4
p ^§§^		0,564	0,943	0,668	0,175
**Sabe escribir**					
Sí	159	3,3 ± 0,5	2,8 ± 0,6	2,9 ± 0,5	3,5 ± 0,4
No	7	3,4 ± 0,7	2,8 ± 1,2	3,0 ± 0,5	3,7 ± 0,4
p ^§§^		0,777	0,843	0,790	0,311
**Programa de Medicina Preventiva**					
Sí	49	3,4 ± 0,4	3,0 ^||||^ ± 0,6	3,0 ± 0,5	3,6 ± 0,4
No	117	3,3 ± 0,5	2,7 ± 0,6	2,9 ± 0,5	3,5 ± 0,4
p ^§§^		0,292	0,015	0,539	0,092
**Tiempo de diagnóstico ECNT** ^⁋⁋^ **(años)**					
Hasta 1 año	19	3,2 ± 0,4	2,6 ± 0,4	2,8 ± 0,5	3,4 ± 0,4
1 a 5	34	3,2 ± 0,5	2,7 ± 0,7	3,0 ± 0,5	3,5 ± 0,4
5 a 14	43	3,4 a ^||||^ ± 0,5	3,0 ± 0,6	3,0 ± 0,6	3,5 ± 0,4
Más de 14	70	3,4 a ^||||^ ± 0,5	2,8 ± 0,7	2,9 ± 0,4	3,5 ± 0,5
p ^††^		0,044	0,106	0,410	0,834

*HPS = Comprensión y apoyo de los profesionales de la salud; ^†^HSI = Información suficiente para cuidar su salud; ^‡^AMH = Cuidado activo de la salud; ^§^SS = Apoyo social a la salud; ^||^HLQ = *Health Literacy Questionnaire*; ^¶^Media ± DE = Desviación estándar; **Análisis de varianza (unidireccional) – *post hoc* Bonferroni; “a” Puntaje medio significativamente más alto que las otras medias del dominio específico; “b” Puntaje medio intermedio, que difiere significativamente tanto de la media más alta como de la media más baja; “c” Media significativamente más baja que las otras medias del dominio específico; ^††^r(p) = Correlación de Pearson; ^‡‡^Diferencia estadísticamente significativa del 1%, estimada mediante la prueba de Análisis de Varianza (Unidireccional) – *post hoc* Bonferroni; ^§§^Prueba t-Student para grupos independientes; ^||||^Diferencia estadísticamente significativa del 5%; ^⁋⁋^ECNT = Enfermedades crónicas no transmisibles

Al comparar los grupos de edad con las escalas Comprensión y Apoyo de (HPS); Información Suficiente para el Cuidado de la Salud (HIS); Atención Activa de la Salud (AMH); Apoyo Social para el Apoyo de la Salud (SS), los grupos de edad se compararon en términos de las escalas de la parte 1, sin diferencias estadísticamente significativas (Tabla 3).

Al comparar la misma escala, fue posible identificar puntuaciones más altas en el grupo con hasta la educación primaria (p=0,009). No hubo diferencia significativa en las puntuaciones entre los dos niveles superiores de educación. Los resultados observados en las demás escalas mostraron que las diferencias entre las puntuaciones de los diferentes niveles de educación no fueron representativas. No se identificaron diferencias significativas en las características de saber leer o escribir (Tabla 3).

Los pacientes participantes fueron comparados con aquellos que no participaban del programa de Medicina Preventiva, y se encontró una diferencia estadísticamente significativa entre los investigados que formaban parte del programa de Medicina Preventiva, en la escala, Información Suficiente para el Cuidado de la Salud (HSI) (p=0,015). En cuanto a las demás escalas, no hubo resultados representativos (Tabla 3).

Al comparar a los pacientes con respecto al tiempo con la ECNT, se identificó una diferencia estadísticamente significativa en los pacientes con diagnóstico entre 5 y 14 años y con más de 14 años, con puntajes medios más altos en la escala comprensión y apoyo de los profesionales de la salud (HPS) (p. =0,044) (Tabla 4).

**Tabla 4 -:** Media y desviación estándar de las escalas CA*, AE^†^ y NHS^‡^ de la escala HLQ^§^, según las características sociodemográficas. Ijuí, RS, Brasil, 2023

**Características**	**N**	**HLQ** ^§^		
		CA*	**AE** ^†^	**NHS** ^‡^
		**Media ± DE** ^||^	**Media ± DE** ^||^	**Media ± DE** ^||^
**Franja etaria (años)**				
Hasta 49	17	2,8a ^¶^ ± 0,6	3,8 ± 0,9	3,5 ± 1,0
De 50 a 69	52	2,8 a ^¶^ ± 0,6	3,9 ± 0,8	3,9 a** ± 0,8
Más de 69	97	2,3 ± 0,7	3,8 ± 0,9	3,4 ± 1,0
p ^††^		<0,001	0,697	0,018
**Edad (años) -** r **(p)** ^‡‡^		-0,301 (p<0,001)	-0,049 (0,529)	-0,133 (p=008)
**Educación**				
No estudió/ Educación primaria	92	2,2 ± 0,7	3,8 ± 0,9	3,4 ± 1,0
Educación secundaria	40	2,7 b ^¶^ ± 0,6	3,9 ± 0,8	3,7 ± 0,9
Educación Superior	34	3,0 a ± 0,6	4,0 ± 0,9	3,8 ± 0,8
p ^††^		<0,001	0,667	0,094
**Sabe leer**				
Sí	158	2,5** ± 0,7	3,9 ± 0,9	3,5 ± 1,0
No	8	1,9 ± 0,8	4,1 ± 0,7	3,7 ± 1,2
P ^§§^		0,010	0,532	0,594
**Sabe escribir**				
Sí	159	2,5** ± 0,7	3,8 ± 0,9	3,5 ± 1,0
No	7	1,9 ± 0,9	4,2 ± 0,6	4,1 ± 0,7
p ^§§^		0,033	0,296	0,129

*CA = Evaluación de la información de salud; **†**AE = Capacidad para interactuar activamente con los profesionales de la salud; **‡**NHS = Navegar en el sistema de salud; ^§^HLQ = *Health Literacy Questionnaire*; ^||^Media ± DE = Desviación estándar; ^¶^Diferencia estadísticamente significativa del 1%, estimada pelo teste de Análisis de varianza (unidireccional) – *post hoc* Bonferroni; **Diferencia estadísticamente significativa del 5%; ^††^Análisis de varianza (unidireccional) – *post hoc* Bonferroni; “a” Puntaje medio significativamente más alto que las otras medias del dominio específico; “b” Puntaje medio intermedio, que difiere significativamente tanto de la media más alta como de la media más baja; “c” Media significativamente más baja que las otras medias del dominio específico; ^‡‡^r(p)Correlación de Pearson; ^§§^Prueba t-Student para grupos independientes

En la Tabla 5, al comparar las franjas etarias, se identificó diferencia estadísticamente significativa en las escalas evaluación de la información de salud (CA) (p<0,001). Se identificó un puntaje medio más alto en las franjas etarias hasta 49 años y de 50 a 69 años. Según la definición de la escala CA alto, significa que la persona es capaz de identificar buena información y fuentes confiables, resolver información contradictoria por sí sola o con la ayuda de otras personas, y esta capacidad estuvo presente en los encuestados en las franjas etarias hasta 49 años y de 50 a 69 años.

La edad de los encuestados también se comparó con los dominios que guiaron las comparaciones en la Tabla 4, y se detectó una correlación estadísticamente significativa y negativa en el dominio CA (r = -0,301; p<0,001), lo que indica que los puntajes altos en el dominio evaluación de la información de salud deben estar correlacionados con las edades más bajas de la muestra (Tabla 5).

La escala navegación en el sistema de salud (NHS) (p=0,018) también se destacó con resultado significativo, con puntaje medio en la franja etaria de 50 a 69 años significativamente superior que el de los demás. En la escala capacidad para interactuar activamente con los profesionales de la salud (AE), no hubo resultados significativos. En cuanto a los resultados de las correlaciones entre la edad y las escalas AE y NHS, no parecen ser representativos. Considerando la comparación entre la educación y las escalas, se identificó diferencia estadísticamente significativa en el dominio CA (p<0,0001).

Los encuestados con niveles de educación secundaria y superior presentaron puntajes medios significativamente más altos que los que tenían un nivel educativo inferior. En las escalas AE y NHS no se presentaron diferencias significativas.

Con respecto al hecho de saber leer, la diferencia significativa se destacó exclusivamente en la escala CA (2,5±0,7 vs. 1,9±0,9 p=0,010), donde el grupo que declaró saber leer presentó un puntaje medio significativamente mayor que el de los demás.

Un resultado similar se observó en relación a la variable saber escribir, al comparar las escalas. La única diferencia significativa se presentó en el dominio CA (p=0,032), lo que indica que los encuestados que declararon saber escribir obtuvieron un puntaje medio significativamente mayor.

**Tabla 5 -:** Media y desviación estándar de las escalas FHI* y UHI^†^ de la escala HLQ^‡^, según las características sociodemográficas. Ijuí, RS, Brasil, 2023

**Características**	**N**	**HLQ** ^‡^	
		FHI*	**UHI** ^†^
		**Media ± DE** ^§^	**Media ± DE** ^§^
**Franja etaria (años)**			
Hasta 49	17	3,5 a ^¶^ **± 1,1**	3,5 a ^¶^ **± 1,1**
De 50 a 69	52	3,4 ^¶^ **± 0,8**	**3,5** ^¶^ **± 0,7**
Más de 69	97	2,9 **± 1,0**	3,0 **± 0,9**
p ^||^		0,002	0,001
**Edad (años) -** r (p)**		-0,334 (p<0,001)	-0,301 (p<0,001)
**Educación**			
No estudió/ Educación primaria	92	2,8 **± 0,9**	3,0 **± 0,9**
Educación secundaria	40	3,5 a ^¶^ **± 0,9**	3,3 b ^¶^ **± 0,8**
Educación Superior	34	3,8 b **± 0,8**	3,6 a ^¶^ **± 1,0**
p ^||^		**<** 0,001	0,002
**Sabe leer**			
Sí	158	3,2 ^††^ **± 1,0**	3,2 ^††^ **± 0,9**
No	8	2,4 **± 0,7**	2,0 **± 0,5**
p ^‡‡^		0,008	**<** 0,001
**Sabe escribir**			
Sí	159	3,2	3,2 ^††^
No	7	2,5	2,0
p ^‡‡^		0,071	0,001

*FHI = Capacidad para encontrar buena información de salud; ^†^UHI = Comprender la información de salud lo suficientemente bien como para saber qué hacer; ^‡^HLQ = *Health Literacy Questionnaire*; ^§^Media ± DE = Desviación estándar; ^||^Análisis de varianza (unidireccional) – *post hoc*; Para resultados con una diferencia estadísticamente significativa, se usa la notación: “a” Puntaje medio significativamente más alto que las otras medias del dominio específico; “b” Puntaje medio intermedio, que difiere significativamente tanto de la media más alta como de la media más baja; “c” Media significativamente más baja que las otras medias del dominio específico; ^¶^Diferencia estadísticamente significativa del 1%, estimada mediante la prueba de Análisis de Varianza (Unidireccional) – *post hoc* Bonferroni; **r(p)= Correlación de Pearson; ^††^Diferencia estadísticamente significativa del 5%; ^‡‡^Prueba t-Student para grupos independientes

Considerando los análisis que involucraron las comparaciones por franjas etarias, en la escala capacidad para encontrar buena información de salud (FHI), hubo una diferencia estadísticamente significativa (p=0,002), lo que indica que los puntajes medios en las franjas etarias hasta 49 años y de 50 años a 59 años fueron significativamente mayores. Se obtuvo un resultado similar al comparar la franja etaria con la escala comprender la información de salud lo suficientemente bien como para saber qué hacer UHI (p=0,001).

Según la definición de la escala, hay evidencia de que las franjas etarias hasta 49 años y de 50 a 69 años tienen una mayor capacidad para buscar información. Y ambas franjas etarias también tienen una mayor capacidad para comprender información escrita sobre salud.

Al correlacionar las escalas con la edad, se identificaron correlaciones estadísticamente significativas y negativas, tanto entre la edad y el dominio FHI (r = -0,334; p<0,001), como con los datos de la escala UHI (r = -0,301; p<0,001). Hay evidencia de que hay correlación entre los encuestados con edades más avanzadas y los puntajes más bajos.

En cuanto a las comparaciones sobre saber leer, se detectaron diferencias estadísticamente significativas en los puntajes de las escalas FHI y UHI. Se identificó que el puntaje medio fue significativamente mayor en los encuestados que declararon saber leer FHI (p=0,010). Un resultado similar se obtuvo al comparar la variable sabe leer. Considerando las comparaciones con la escala UHI, en la pregunta saber escribir (p=0,010), se observó diferencia estadísticamente significativa exclusivamente en este dominio, lo que indica mayor puntaje medio en el grupo que informó que sabe escribir.

## Discusión

Este es el primer estudio brasileño que evaluó la AS de personas que viven con ECNT y comprobó la relación con variables sociodemográficas (edad, escolaridad, saber leer y escribir y si forma parte de la medicina preventiva) y clínicas (tiempo de diagnóstico de la ECNT), mediante análisis bivariado. Se identificó que los puntajes más altos se observaron en las escalas apoyo social a la salud y capacidad para interactuar activamente con profesionales de la salud, ambos resultados similares a los de otro estudio internacional con personas que viven en una comunidad pesquera rural en el norte de Egipto ^( 13 )^.

Predominaron las personas con ECV, resultado similar al de un estudio que evaluó la AS en Brasil con personas afectadas por ECNT, en el que predominó la ECV con 230 personas (29%) y sexo femenino (67,5%) ^( 10 )^. Otros estudios actuales también confirman estos resultados ^( 1 , 6 )^. Se observó una reducción de la AS con el aumento de la edad y menor educación. La AS juega un papel importante en el manejo de las ECNT, consideradas complejas, que representan un desafío importante y a largo plazo para las personas y el sistema de salud ^( 4 , 6 )^.

Los puntajes más bajos se encontraron en las escalas “evaluación de la información de salud” y “capacidad para encontrar buena información de salud”, lo que coincide con otros estudios brasileños que utilizaron el mismo instrumento con personas que viven con VIH y con enfermedad renal crónica ^( 12 , 14 )^.

En la presente investigación, personas con edades avanzadas y baja escolaridad tuvieron mejores desempeños en el dominio “apoyo social a la salud”, resultado similar al encontrado en otros estudios ^( 15 )^. Además, un apoyo social de calidad promueve mejores resultados de salud ^( 16 )^ y mejora la autogestión ^( 17 )^. Por ende, brindarle educación y apoyo a los familiares y cuidadores insertos en este contexto puede ser una estrategia útil para los servicios de salud, ya que otros estudios ^( 18 - 19 )^ sugieren que los cuidadores de personas con ECNT necesitan información sobre el cuidado, y los principios de la AS son cruciales en la vida diaria de los cuidadores, profesionales y gerentes para obtener mejores resultados y tomar decisiones de salud ^( 11 )^.

Un estudio realizado en Australia muestra que pacientes con edades avanzadas y menor nivel educativo necesitan y/o reciben mayor apoyo social a la salud, lo que favorece la AS en esta población ^( 20 )^. Otra investigación en Brasil obtuvo resultados similares a los de este estudio ^( 14 , 21 )^.

Es fundamental que los profesionales de la salud evalúen el nivel de AS de los pacientes y adapten la información y el apoyo a las competencias de AS de acuerdo con el contexto de sus pacientes, de forma individual, dado que eso favorece el manejo de la condición crónica de salud a lo largo de su vida ^( 9 )^. Cabe destacar que las estrategias basadas en la comunicación asertiva, la información comprensible y el acceso claro a los servicios favorecen la AS de las personas ^( 7 , 22 )^.

Datos de este estudio revelan que las personas que formaban parte de un programa de medicina preventiva tuvieron una mejor media en el dominio información suficiente para cuidar su salud. Es decir, se sentían seguros de tener la información que necesitaban para vivir, manejar sus condiciones y tomar decisiones sobre su salud ^( 8 )^, a diferencia de otros estudios que presentaron medias más bajas ^( 12 - 13 )^, lo que destaca la importancia de los programas que se basan en los principios de la AS ^( 3 )^.

Es importante que el paciente tenga relación con al menos un profesional de la salud que lo conozca bien y en quien pueda confiar para recibir consejos e información útil que le ayude a comprender las indicaciones y tomar decisiones sobre su salud ^( 8 )^. Fortalecer el vínculo facilita la continuidad de la atención y brinda oportunidades para el desarrollo de estrategias de manera específica. Se considera estratégico que los profesionales de la salud transmitan información de manera clara y con lenguaje accesible, para favorecer la comprensión y fomentar la autogestión ^( 23 )^, dado que los servicios de salud son la principal puerta de entrada y se los considera fuente segura de información para los usuarios.

Se encontró que los participantes en el presente estudio demostraron debilidades en las escalas información suficiente para cuidar su salud y evaluación de la información de salud. Presentaron lagunas en el conocimiento sobre salud, no lograron comprender la información que recibían y creían que no tenían la información necesaria para la autogestión, lo mismo se observó en otro estudio ^( 12 )^ que identificó un nivel bajo/insuficiente de adherencia al tratamiento farmacológico en las personas con VIH, debido a las debilidades en dichas escalas.

Estos resultados tienen implicaciones importantes y confirman que es necesario y que hay que avanzar en la atención a las personas con ECNT, en términos de comunicación asertiva entre pacientes y profesionales de manera específica, como lo demuestran otros estudios con resultados similares ^( 11 , 24 - 25 )^. El acceso a la información no es el principal problema, sino la calidad, ya que las personas necesitan entender claramente las indicaciones para la autogestión. Para ello, se pueden adoptar recursos como entregar material educativo sobre la enfermedad y el tratamiento en un lenguaje accesible y de una fuente segura ^( 7 , 23 )^.

La comunicación asertiva se puede utilizar mediante el empleo de la técnica *teach back*, que favorece la comprensión de la información ^( 26 )^. Estas estrategias ofrecen apoyo a las personas para aumentar el conocimiento, la capacidad para tomar decisiones compartida, el compromiso y la autogestión ^( 26 - 27 )^.

El presente estudio identificó que las personas más jóvenes con un mayor nivel de educación tenían potencialidad de AS para identificar buena información en fuentes confiables, así como para resolver información contradictoria por sí solas o con la ayuda de otras personas. El nivel de educación es relevante, ya que se sabe que las personas con mayor nivel de educación pueden tener mejor acceso a información sobre la atención de la salud y el tratamiento de las ECNT ^( 28 )^. Esto, si bien influye, no siempre se considera una característica determinante, dado que personas con altos niveles de estudio pueden tener una AS deficiente, porque esta es específica y va más allá de saber leer o escribir, incluye las habilidades para tomar decisiones, promover la salud, acceder y navegar en el sistema de salud ^( 7 )^.

El dominio cuidado activo de la salud también presentó debilidades para la AS. Se relaciona con ser responsable de su salud y participar proactivamente para tomar sus propias decisiones. Este hecho puede estar asociado con la edad y el bajo nivel educativo, ya que la edad avanzada puede reducir la capacidad para realizar tareas cognitivas que requieren procesamiento de información. Las personas mayores parecen tener más dificultades para completar tareas que requieren razonar o sacar conclusiones a partir de la información presentada ^( 29 )^.

Con respecto a ese tema, los autores señalan que los profesionales de la salud deben preocuparse por el apoyo social, especialmente los enfermeros, con el fin de mejorar las prácticas educativas que puedan brindar una escucha calificada, aclaraciones sobre el cuidado e inclusive considerar cuestiones emocionales ^( 30 )^, para abordar de forma integral a los adultos mayores y con mayor vulnerabilidad.

Considerando los dominios capacidad para encontrar buena información de salud y comprender la información de salud lo suficientemente bien como para saber qué hacer, se identificó que, hasta los 69 años, las personas mostraban potencialidad de AS. Las personas deben poder buscar, comprender y utilizar diferentes fuentes de información, y ser capaces de escribir adecuadamente en formularios cuando sea necesario.

El estudio permitió identificar aspectos que hay que mejorar en la atención, para contribuir al desarrollo de estrategias y favorecer la AS. Gracias al estudio, se pudo notar que es fundamental invertir en políticas de salud, en el ámbito público y privado, que se basen en estrategias de AS, y que también es necesario apoyar a los gestores, profesionales de la salud y usuarios comprometidos para que el cambio ocurra.

Además, los resultados pueden contribuir a otros estudios publicados y en curso a nivel universal, y facilitar la reflexión sobre la AS en las prácticas de gestión en salud en Brasil y otros países.

Este estudio tiene algunas limitaciones, el tipo de estudio elegido, cuantitativo y transversal, que no permite establecer una relación causal entre las variables, además de realizarse en un solo hospital general. Por tanto, se debe tener cuidado a la hora de generalizar los resultados. A pesar de las limitaciones, este estudio trae innovaciones sobre la importancia de considerar las LS para la calificación de la atención en salud a las ENT. Los resultados pueden brindar apoyo para futuras investigaciones que investiguen la relación causal entre LS y variables sociodemográficas y clínicas en pacientes hospitalizados con ENT.

## Conclusión

Los pacientes mostraron potencialidad de AS, en las escalas apoyo social (3,5±0,4), capacidad para interactuar con los profesionales de la salud (3,9±0,9) y navegar en el sistema de salud (3,6±1,0); por otro lado, presentaron debilidades en la evaluación de los servicios (2,5±1,0) y en la capacidad para encontrar buena información de salud (3,1±1,0).

Hubo asociación significativa entre AS y variables sociodemográficas, como edad, educación y saber leer y escribir. Los pacientes hasta 49 años y de 50 a 59 años tenían medias más bajas en la escala apoyo social a la salud, y aquellas con menor educación tenían medias más altas. Las franjas etarias más jóvenes mostraron mejores resultados de AS en las escalas evaluar los servicios de salud, capacidad para encontrar buena información de salud y comprender la información de salud lo suficientemente bien como para saber qué hacer.

Los pacientes más jóvenes, con educación superior y habilidades de lectura y escritura presentaron puntajes más altos en escalas relacionadas con la evaluación y comprensión de la información de salud. Por otro lado, los individuos con mayor edad y menor educación presentaron puntajes más bajos, lo que indica debilidades en la comprensión de la información de salud, pero se identificó que la escala apoyo social a la salud mostró potencialidad y podría favorecer la AS en la evaluación y utilización de los servicios de salud.

Las personas que formaban parte del programa de medicina preventiva tuvieron medias más altas en la escala información suficiente para cuidar su salud. Las personas que habían padecido ECNT durante un período más prolongado sentían que los profesionales de la salud las comprendían y apoyaban más.

Por lo tanto, es necesario que los profesionales de la salud sean capaces de reflexionar e implementar estrategias de AS de manera específica para personas de mayor edad y bajo nivel educativo, para que puedan tomar decisiones sobre su salud y bienestar.
